# Immersive Virtual Reality Training to Improve Novice Physicians’ Emergency Response Skills: Randomized Controlled Trial

**DOI:** 10.2196/71455

**Published:** 2026-03-19

**Authors:** Yeon-Ju Huh, Ju Whi Kim, Narae Yoon, Seoi Jeong, Hyoun-Joong Kong, Sun Jung Myung

**Affiliations:** 1 Office of Medical Education, Seoul National University College of Medicine, Education building, 2nd Fl., 103, Daehak-ro, Jongno-gu, Seoul, 03080, Republic of Korea, 8227408177, 8227411187; 2 Department of Surgery, Seoul National University Hospital, Seoul, 03080, Republic of Korea; 3 Department of Pediatrics, Seoul National University Hospital, Seoul, Republic of Korea; 4 Department of Psychiatry, Seoul National University Hospital, Seoul, Republic of Korea; 5 Department of Transdisciplinary Medicine, Seoul National University Hospital, Seoul, Republic of Korea; 6 Department of Clinical Medical Sciences, Seoul National University College of Medicine, Seoul, Republic of Korea; 7 Institute of Convergence Medicine with Innovative Technology, Seoul National University Hospital, Seoul, Republic of Korea; 8 Department of Medicine, Seoul National University College of Medicine, Seoul, Republic of Korea; 9 Department of Internal Medicine, Seoul National University Hospital, Seoul, Republic of Korea

**Keywords:** virtual reality, high-fidelity simulation, simulation-based education, medical Interns, emergency response, clinical decision-making, confidence, satisfaction, educational technology, randomized controlled trial

## Abstract

**Background:**

Simulation-based training is essential for preparing medical interns to manage high-stakes emergencies. Although virtual reality (VR)-based simulation has been rapidly integrated into medical education, there remains limited evidence directly assessing its effectiveness relative to established high-fidelity simulation (HFS) methodologies.

**Objective:**

This study aimed to assess the perceived educational effectiveness of VR and HFS in enhancing novice physicians’ confidence, satisfaction, and perceived preparedness for managing acute oxygen desaturation.

**Methods:**

A randomized controlled trial was conducted with 168 medical interns from Seoul National University Hospital. Participants were randomly assigned to VR group (n=81) or HFS group (n=87). Overall, 4 participants were excluded due to incomplete surveys, leaving 164 for analysis (VR: 79 and HFS: 85). Both groups were trained to manage simulated patients with low oxygen saturation. Confidence (10-point Likert scale) and satisfaction (7-point Likert scale) were measured using pre and posttraining surveys. Usability was assessed with the User Experience Questionnaire-Short. Between-group comparisons were conducted using *t* tests and chi-square tests, while within-group confidence changes were analyzed using paired *t* tests and repeated-measures analysis of variance. To account for correlated data and estimate effect sizes, generalized estimating equations were applied, with statistical significance set at *P*<.05. Focus group interviews at 1 and 5 months posttraining explored real-world application and behavior transfer. Transcripts were independently reviewed by 2 researchers (YJH and SJM) and thematically analyzed to identify recurring patterns and insights related to clinical behavior.

**Results:**

Confidence in managing oxygen desaturation significantly improved from a mean 3.78 (SD 2.12) to mean 6.20 (SD 2.02) across VR and HFS groups (*t*_163_=−14.04; *P*<.001), with no significant difference between groups (*F*_1,162_=3.28; *P*=.07). Satisfaction was high overall mean 6.07 (SD 1.02), but significantly greater in the HFS group than in the VR group (mean 6.23, SD 0.92 vs mean 5.89, SD 1.10; *t*_162_ =2.29; *P*=.02). HFS participants rated tutor guidance (mean 6.49, SD 0.86 vs mean 6.10, SD 1.02; *P*=.008) and authenticity (mean 6.24, SD 1.05 vs mean 5.77, SD 1.15; *P*=.006) higher, whereas both groups scored usability above 5 on all items. Qualitative analyses revealed complementary strengths. Interns valued VR for its immersive environment, focused repetition, and reduced distractions that facilitated stepwise problem-solving. HFS was praised for palpable realism, hands-on practice with equipment, and immediate feedback that reinforced team communication and role clarity. Across follow-up interviews, interns reported improved recognition of desaturation, more structured initial responses (airway assessment, oxygen delivery adjustments, and escalation), and greater willingness to act promptly under pressure—suggesting perceived transfer of learning to clinical practice beyond the simulation lab.

**Conclusions:**

VR may complement HFS in emergency response training. Both modalities were associated with improvements in interns’ self-reported confidence and perceived preparedness. The scalability and accessibility of VR suggest its potential value in diverse training contexts.

## Introduction

The ability to respond effectively to emergencies is a critical competency for medical professionals. Ensuring that interns and new practitioners are adequately prepared for these high-stakes scenarios is essential for improving patient outcomes and reducing errors [1]. A rapid decline in oxygen saturation represents one of the most critical and life-threatening challenges in medical practice, demanding immediate intervention to avert severe complications or death. This emergency is particularly prevalent in high-stakes settings, such as intensive care units, emergency departments, and operating rooms, where the ability of physicians to respond swiftly and effectively is crucial for patient survival and recovery. However, for novice doctors with limited clinical experience, managing such crises can be particularly daunting.

Traditional educational approaches for emergency response training, such as didactic lectures and case-based discussion, remain the predominant methods used in many medical curricula [2,3]. While foundational, these approaches may offer limited opportunities for immersive, hands-on experience necessary to equip learners fully for real-world challenges. They rarely replicate the stress, urgency, and unpredictability of real-life emergencies. Simulation-based education helps address this gap by providing a safe and controlled environment for developing and practicing skills [4,5]. High-fidelity simulation (HFS) using advanced mannequins to mimic realistic patient responses provides an interactive learning experience with immediate feedback without patient risk, but it is resource-intensive, requiring substantial investment and logistical support [6,7]. Recent advances in technology have introduced virtual reality (VR) as a promising alternative to traditional HFS [8]. VR simulation leverages immersive, computer-generated environments to recreate highly realistic clinical scenarios. VR offers several benefits, including lower cost, greater accessibility, and the ability to simulate a wide range of scenarios that might be impractical or impossible to replicate with physical mannequins [9]. However, the comparative effectiveness of VR and HFS in training medical interns for emergency response remains underexplored [10]. In addition, most medical VR systems focus on step-by-step procedures training rather than dynamic decision-making under pressure [11-13].

This study compared VR and HFS in training novice physicians for emergency oxygen desaturation management. Beyond immediate satisfaction and confidence (Kirkpatrick Levels 1 and 2), we explored the long-term perceived clinical transfer (Kirkpatrick Level 3) by examining learners’ reflections on their responses to similar clinical situations 1 and 5 months posttraining [14]. This longitudinal perspective aligns with Knowles’ adult learning theory [15], emphasizing the transition from cognitive readiness to the practical application of experiential knowledge. We hypothesized that VR would demonstrate educational outcomes comparable to HFS in terms of both immediate psychological readiness and perceptions of learning transfer, while offering a distinct learner experience.

## Methods

### Study Design

**Figure 1.**
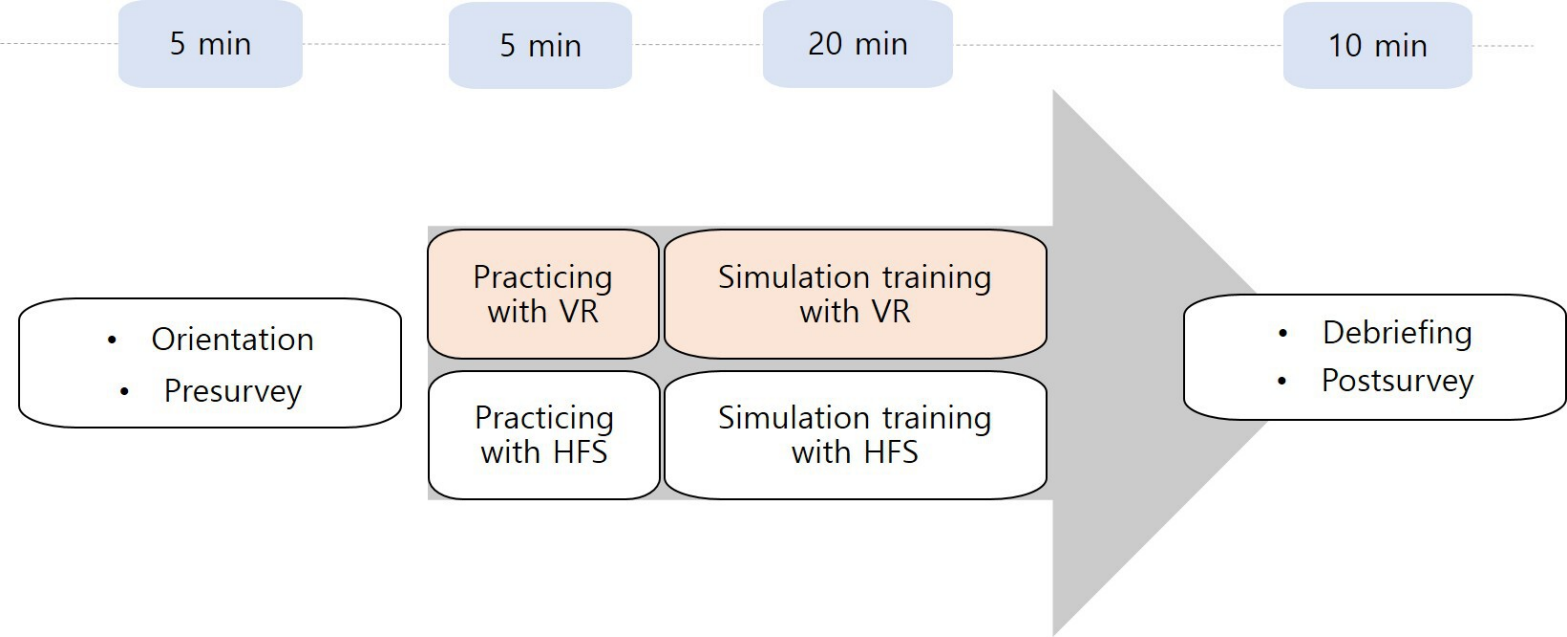
Study flow chart. HFS: high-fidelity simulation; VR: virtual reality.

A 2-arm parallel randomized controlled trial was conducted to compare the educational effectiveness of VR and HFS methods. The sample size for this study was based on a prior study using G*Power 3.1.2 software (Heinrich Heine University Düsseldorf) for an independent *t* test, assuming a type I error (α) of .05, statistical power (1–*β*) of .80, and an effect size (Cohen d) of 0.80, which indicated that a minimum of 52 participants would be required [16]. The random allocation sequence was generated using the clinical trial randomization tool [17] by an independent third party unrelated to the study, ensuring reproducibility. To ensure allocation concealment, the randomization list was stored on a password-protected server accessible only to the independent administrator. All participant data were anonymized, and the trial administrators and personnel analyzing the data were blinded to the participants’ identities and group assignments. Participants simulated scenarios involving desaturation based on their previous knowledge using the specific training method for their group, followed by a debriefing session to reinforce learning. Following completion of the simulation scenarios, participants in both groups engaged in an in-person debriefing session facilitated by faculty instructors (Figure 1). Debriefing sessions were conducted using a structured format with comparable group size and time allocation across the VR and HFS groups. Tutors were faculty physicians with clinical experience in acute care and prior experience in simulation-based medical education. All tutors had previously facilitated HFS sessions and received orientation to the study scenarios and debriefing framework before the intervention. Tutors facilitated the simulation sessions and led the postscenario debriefing. The findings were reported following the CONSORT (Consolidated Standards of Reporting Trials) guidelines, [18] the checklist and the trial protocol can be accessed in Checklist 1.

### Participants and Recruitment

Participants were postgraduate year 1 medical interns who had recently graduated from medical school and were about to begin their internship training at a tertiary teaching hospital. Eligible interns were identified from the annual intern cohort and were invited to participate in February 2024 through email invitations and in-person announcements during scheduled educational sessions. Interns who declined participation or were unable to attend the scheduled simulation sessions were excluded. Participation was voluntary, and interns were explicitly informed that participation or nonparticipation would not affect their training, evaluation, or professional standing. To minimize potential coercion, recruitment and consent procedures were conducted separately from routine educational assessments, and participation status was not disclosed to supervising faculty involved in intern evaluation. Written informed consent was obtained from all participants prior to enrollment. After consent, participants were assigned identification numbers and randomly allocated to either the VR or HFS training group using simple randomization.

### Development of an Educational Program

#### Scenario Development

The goal of the program is to enable participants to quickly recognize when a patient’s oxygen saturation is dropping, identify the critical factors that need to be assessed, take appropriate action to manage the patient, and promptly recognize when it is necessary to seek additional help.

The simulation program was designed to alter the patient’s condition based on the interns’ expected responses to critical aspects, such as a disconnected monitor or oxygen line, low oxygen flow or low blood pressure, and when oxygen saturation dropped. To address the need for flexible problem-solving in emergency situations, we developed a scenario-based VR system that allows nonlinear decision-making. Unlike procedural-focused VR simulations, our system presented participants with complex, evolving emergency scenarios that required rapid assessment and adaptable responses. The simulation scenario was refined and enhanced through research team meetings to ensure that it was properly aligned with the educational objectives.

The program included simple and complex scenarios to promote learning through experience. The first scenario involved a patient whose condition was stable, with the primary issue being a disconnected oxygen line resulting in a lower oxygen saturation level. Resolving this issue by reconnecting the oxygen line restored the patient’s oxygen saturation to the normal level. The second scenario presented a more challenging situation where both the oxygen line and saturation probe were initially disconnected. Despite reconnecting the oxygen line and saturation probe, there was no improvement in the patient’s heart rate or consciousness. This lack of clinical improvement signaled ongoing deterioration despite correction of equipment-related issues, reflecting a critical condition potentially related to worsening of underlying medical problem. In this scenario, the patient remained unstable and required further escalation of care, including activation of the cardiopulmonary resuscitation team.

### Development of the VR Simulation Training Program

The VR program was developed using the Unity Technologies platform (Unity Software, Inc.) and used immersive VR devices, including the Oculus Quest Pro, the Oculus Quest 3, and the Oculus Quest 2 (Meta Platforms, Inc.). The virtual environment consisted of a hospital hallway, the patients, oxygen tanks, monitors, and saturation probes, which were meticulously crafted using real-time 3D rendering and 3D modeling techniques. This setup provided a first-person perspective, allowing participants to engage in realistic interactions and auditory experiences as if they were physically present in the environment.

**Figure 2.**
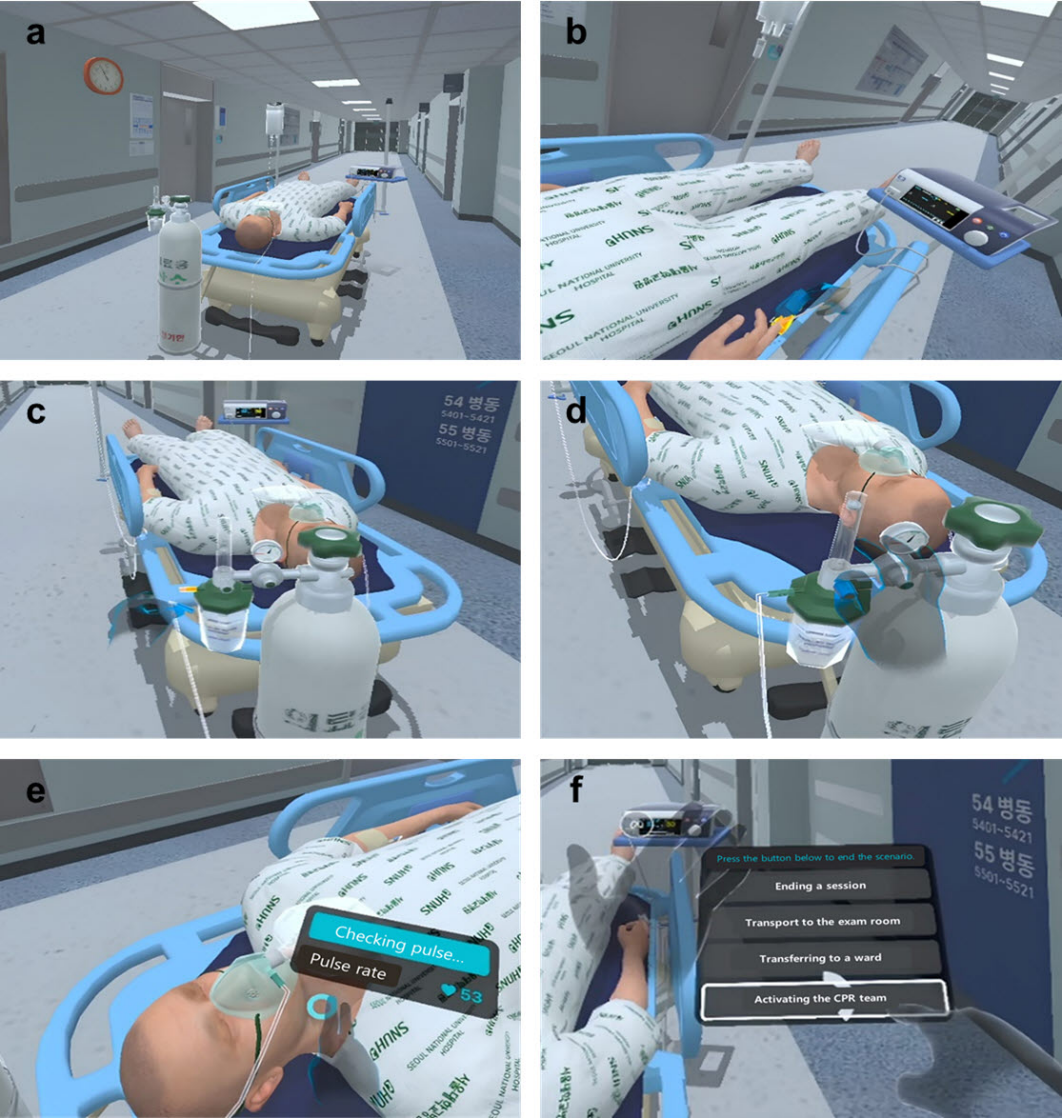
Scenes captured from a virtual reality simulation program showing how to manage a patient in desaturation. (a) The participant encounters an urgent situation where a monitor alarm sounds during a patient transfer; (b) after confirming that no numbers are displayed on the monitor, the participant places the saturation probe back on securely; (c) if oxygen saturation does not increase, the participant securely attaches the disconnected oxygen line; (d) the participant maximizes the oxygen flow rate; (e) the participant checks the patient’s level of consciousness and pulse rate; and (f) the participant chooses a plan depending on whether the patient is recovering, such as calling for help (eg, activating a cardiopulmonary resuscitation team) or proceeding to the exam room as scheduled. CPR: cardiopulmonary resuscitation.

The VR scenario focused on training participants to manage patients with suddenly decreasing oxygen saturation, particularly when immediate assistance was not readily available. Participants were required to prioritize checks (eg, oxygen line connectivity, oxygen flow rate, and the patient’s level of consciousness) and manage the situation accordingly. The system was designed to facilitate direct interaction, enabling participants to identify and resolve these issues effectively. Once the participants believed they had completed all of the necessary actions, they were prompted to determine the next appropriate step, such as transferring the patient to the laboratory, transferring them to the ward, or calling the cardiopulmonary resuscitation team (Figure 2).

### Conventional SimMan ALS Simulation Program

An educational program based on the scenarios developed was programmed into the HFS SimMan ALS simulator (Laerdal Medical). Situations involving an intentionally disconnected O_2_ line and saturation monitor probe were manually staged. Vital signs corresponding to these scenarios were programmed and displayed through the SimMan ALS simulator. The intervention required participants to implement appropriate responses to restore the patient’s vital signs, which was considered a successful intervention.

### Program Evaluation

To evaluate the educational impact and learner experience, we used a combination of standardized and study-specific instruments focusing on 3 primary constructs: self-efficacy (confidence), learner satisfaction, and perceived authenticity.

#### Self-Efficacy (Confidence)

The primary outcome was the change in self-reported confidence in managing oxygen desaturation, measured on a 10-point Likert scale [19] To ensure the specificity of the training effect, we included “control questions” regarding unrelated emergencies (eg, seizures and anaphylaxis). This allowed us to distinguish between a general increase in confidence and the specific cognitive gains from the simulation.

#### Study-Specific Questionnaires

Secondary outcomes, including learner satisfaction and perceived educational experience (tutor guidance, training duration), were assessed using questionnaires developed by the research team. These items were adapted from routinely administered institutional course evaluation protocols used in our tertiary teaching hospital to support content relevance.

#### Perceived Authenticity and Usability

Perceived authenticity was measured using a 7-point Likert scale [20] to evaluate how well each modality mirrored clinical reality—a particularly relevant factor for novice learners. Usability and VR-specific symptoms (eg, motion sickness) were assessed using the User Experience Questionnaire-Short (UEQ-S), a validated 7-point scale [21].

#### Design and Validity

All study-specific instruments were reviewed by a panel of 3 senior medical education experts (YJH, JWK, and SJM) to support face and content validity regarding the target clinical scenarios. While formal psychometric factor analysis was not performed, the items were designed to descriptively capture the immediate psychological and educational reactions of the interns, consistent with Kirkpatrick Level 1 and 2 evaluations.

To further evaluate the effect of the program, qualitative data were collected through follow-up focus group interviews (FGIs), as detailed below.

### Qualitative Methods and Mixed-Methods Integration

We used an embedded qualitative approach within the randomized controlled trial to explore participants’ perceptions of learning transfer to clinical practice (Kirkpatrick level 3) [14] and to contextualize the quantitative outcomes. Reporting was guided by established qualitative reporting standards (eg, COREQ), with adaptations appropriate for an embedded mixed-methods design.

### Sampling and Recruitment

Interns from both trial arms and tutors who facilitated the sessions were eligible. We invited eligible participants after the training and again at follow-up time points; participation was voluntary. FGIs were conducted at 1 month (interns and tutors) and 5 months (interns only) posttraining. At 1 month, 5 interns (VR=3; HFS=2) and 4 tutors participated; at 5 months, 4 interns (VR=2; HFS=2) participated. The number of individuals invited versus those who participated was not systematically recorded in the original study logs.

### Data Collection

Semi-structured interview guides were developed by the research team based on the study objectives, prior simulation literature, and the Kirkpatrick framework. Questions explored perceived preparedness, examples of applying the training in real clinical settings, and perceived strengths and limitations of each modality. FGIs were moderated by a facilitator not involved in participant evaluation, audio-recorded, transcribed verbatim, and deidentified.

### Analysis

Overall, 2 researchers (YJH and SJM) independently coded transcripts using a concise framework-based thematic analysis. An initial coding framework was informed by Kirkpatrick level 3 constructs (eg, recognition of desaturation, initial actions, escalation, and communication), with additional inductive codes added for emergent themes. Coders met to reconcile differences and refine themes through iterative discussion until consensus was achieved. Themes were then mapped to the Kirkpatrick framework to support interpretation as perceived learning transfer, rather than as objectively verified behavior change; given the small and relatively homogeneous sample, the qualitative findings were presented descriptively to illustrate how the training translated into clinical practice, rather than to draw broadly generalizable conclusions.

### Statistical Analysis

The answers from all questionnaires were compared between the VR and HFS groups. The primary outcome was the pre- to posttraining change in self-reported confidence for managing oxygen desaturation. The primary between-group analysis tested the group-by-time interaction for desaturation confidence. Secondary outcomes included overall satisfaction and subdomains, UEQ-S usability scores, and confidence changes for nontargeted scenarios (seizure, anaphylaxis, general care, and general skills). Exploratory analyses included preference data, VR head-mounted display (HMD) log metrics, and the qualitative focus group findings, which were used to contextualize and interpret the quantitative results. Statistical analysis was performed using the SPSS (version 25; IBM Corp) statistical package. Continuous variables were analyzed with the *t* test, and proportions were compared with the chi-square test. Changes in confidence before and after training, as well as in confidence according to the training method, were analyzed using a paired *t* test, repeated-measures analysis of variance, and generalized estimating equations. Effect sizes were calculated using the generalized estimating equations and Cohen *d* values. A *P* value <.05 was considered significant.

### Ethical Considerations

This study received approval from the Seoul National University Hospital Institutional Review Board (IRB no. H2402-076-1511) before commencement and was registered at Clinical Trials official.gov website (no. NCT06295887) on 13 February 2024 [22]. This study was conducted following the Declaration of Helsinki, and informed consent was obtained from all eligible participants. All participant data were anonymized prior to analysis to protect privacy. Confidentiality was maintained through the use of identification codes and secure data storage. No financial compensation was provided for participation.

## Results

### Participants and Baseline Characteristics

**Figure 3.**
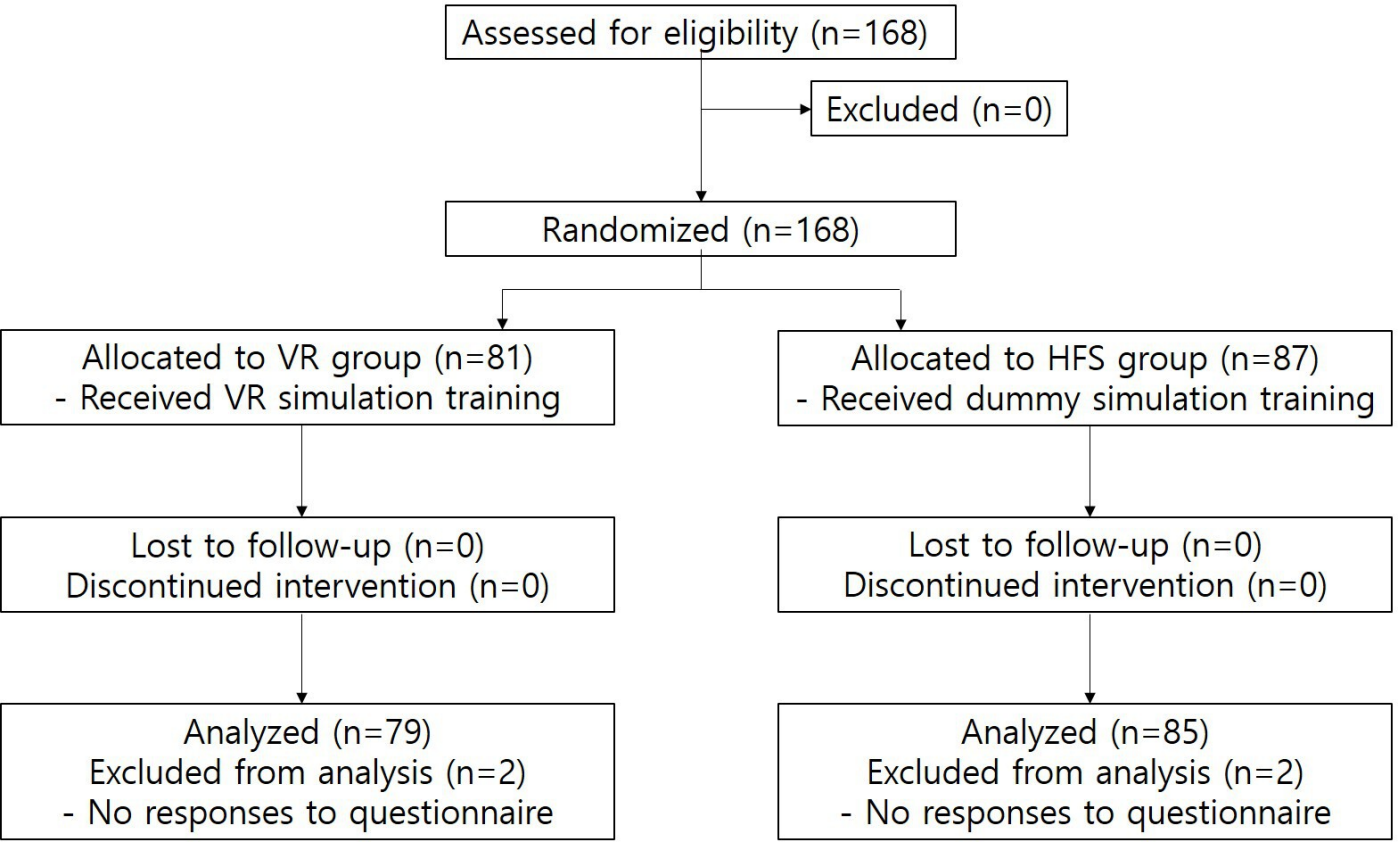
Flow diagram of the two-group parallel randomized trial. HFS: high-fidelity simulation; VR: virtual reality.

This study included 168 prospective interns who were randomly assigned to either the VR group (n=81) or the HFS group (n=87). No dropouts were reported before randomization. Data from 4 participants who did not complete the presurvey were excluded from the final analysis, resulting in 79 participants in the VR group and 85 in the HFS group (Figure 3). The sex ratio (male:female) was 1.9:1 in the HFS group and 1.4:1 in the VR group, with no significant difference between the groups (*P*=.34). The mean age did not differ between the groups (*P*=.29) (Multimedia Appendix 1). All interns had passed the Korean medical licensing examination, meeting a common minimum competence standard, and were selected using comparable pre-entry criteria (eg, medical school grade point average and Korean medical licensing examination written scores).

### Changes in Confidence Before and After Training

On a 10-point scale, the mean (SD) confidence score for managing oxygen desaturation increased significantly from mean 3.78 (SD 2.12) before training to mean 6.20 (SD 2.02) after training (*t*_163_=−14.04; *P*<.001). Notably, confidence in handling unrelated scenarios, such as seizures, anaphylaxis, general care, and basic skills, also improved, suggesting a broader educational impact. However, the increase in confidence for managing oxygen desaturation was significantly higher than other situations (*t*_163_=−13.87; *P*<.001; Cohen *d*=−1.08).

As shown in Table 1, both groups demonstrated significant improvements: from mean 3.82 (SD 2.31) to mean 6.54 (SD 1.91) in the HFS group (*t*_84_=−10.79; *P*<.001) and from mean 3.72 (SD 1.91) to mean 5.82 (SD 2.08) in the VR group (*t*_78_=−9.15; *P*<.001). The training method did not significantly affect confidence (*F*_1, 162_=3.28; *P*=.07), indicating that we did not detect a statistically significant difference between groups in confidence change within the precision of this study; however, this trial was not designed or powered as a noninferiority study and no noninferiority margin was prespecified. Similarly, confidence in other nontargeted situations also improved in both groups without significant between-group differences.

**Table 1.** Differences in self-confidence between training methods.

Confidence domain	Pretraining (N=164), mean (SD)		Posttraining (N=164), mean (SD)		Sources	*P* value
HFS^a^ (n=85)	VR^b^ (n=79)	HFS (n=85)	VR (n=79)
Seizure	3.67 (2.38)	3.38 (1.89)	4.49 (2.02)	4.32 (2.07)	Group	.54
					Time	<.001
					Group*Time	.53
Desaturation^c^^d^	3.82 (2.31)	3.72 (1.91)	6.54 (1.91)	5.82 (2.08)	Group	.19
					Time	<.001
					Group*Time	.07
Anaphylaxis	4.85 (2.58)	4.67 (2.26)	5.32 (2.08)	5.05 (2.10)	Group	.62
					Time	.004
					Group*Time	.75
General care	4.24 (2.29)	4.18 (1.92)	5.33 (1.88)	5 (1.96)	Group	.59
					Time	<.001
					Group*Time	.41
General skills	4.47 (2.23)	4.14 (1.81)	5.28 (1.87)	4.96 (1.88)	Group	.31
					Time	<.001
					Group*Time	.85

a HFS: high-fidelity simulation.

b VR: virtual reality.

c Rated on a 10-point Likert scale ranging from 1 (strongly disagree) to 10 (strongly agree).

d Result of the simulation training offered.

VR simulation was the most frequently preferred training method (63/139, 45.3%), followed by HFS (54/139, 38.8%) and lectures (22/139, 15.8%). Notably, participants’ preferences tended to correspond to the training method they were assigned, indicating that postassignment exposure may have shaped their reported preferences rather than reflecting preexisting inclinations. However, concordance between a participant’s assigned and preferred training method was not associated with satisfaction (*t*_137_=0.21, *P*=.13) or perceived improvement in confidence following training (*F*_1,137_=0.08, *P*=.45).

### Satisfaction With the Educational Program

Participants in both groups reported high levels of satisfaction with the educational program, with an overall mean (SD) score of 6.07 (1.02) out of 7. As shown in Table 2, the HFS group was more satisfied overall than was the VR group (mean 6.23, SD 0.92 vs mean 5.89, SD 1.10; *t*_162_=2.29, *P*=.02), particularly regarding tutor guidance (mean 6.49, SD 0.86 vs mean 6.10, SD 1.02; *P*=.008) and authenticity of the educational experience (mean 6.24, SD 1.05 vs mean 5.77, SD 1.15; *P*=.006). However, the overall difference between the groups was minimal, and both groups reported very high levels of overall satisfaction.

**Table 2.** Student satisfaction with the simulation education.

Themes^a^	HFS^b^ (n=85), mean (SD)	VR^c^ (n=79), mean (SD)	*P* value
Overall satisfaction	6.23 (0.92)	5.89 (1.10)	.02
Training duration	6.06 (1.21)	5.81 (1.25)	.20
Tutor’s guidance	6.49 (0.86)	6.10 (1.02)	.008
Authenticity	6.24 (1.05)	5.77 (1.15)	.006

a Rated on a 7-point Likert scale ranging from 1 (strongly disagree) to 7 (strongly agree).

b HFS: high-fidelity simulation.

c VR: virtual reality.

The usability was evaluated by the UEQ-S. As shown in Table 3, the analysis of 8 items on a 7-point scale revealed significant differences between the 2 groups on 4 items. The HFS group rated the training as more supportive, easy, efficient, and clear than did the VR group. No significant differences were detected between the groups in terms of excitement, interest, inventiveness, or being cutting-edge. All items had scores above 5, indicating the usability of both training methods.

**Table 3.** Results of User Experience Questionnaire-Short version.

Negative^a^ (1)	HFS^b^ (n=85), mean (SD)	VR^c^ (n=79), mean (SD)	*P* value	Positive^a^ (7)
Obstructive	5.97 (0.96)	5.62 (1.09)	.03	Supportive
Complicated	5.45 (1.13)	5.05 (1.27)	.04	Easy
Inefficient	5.83 (1.00)	5.42 (1.24)	.02	Efficient
Confusing	5.71 (1.31)	5.00 (1.42)	.001	Clear
Boring	6.00 (1.23)	5.96 (1.18)	.84	Exciting
Not interesting	5.99 (1.23)	5.99 (1.20)	.99	Interesting
Conventional	5.33 (1.18)	5.69 (1.30)	.06	Inventive
Usual	5.31 (1.17)	5.67 (1.41)	.08	Leading edge

a Rated on a 7-point Likert scale ranging from 1 (strongly negative) to 7 (strongly positive).

b HFS: high-fidelity simulation.

c VR: virtual reality.

### Analysis of the VR Group Log Data

Further analysis was conducted using the log data recorded from the VR group on a HMD. Usable data were available for 32 simple and 39 complex cases, with 29 participants having both. The logs captured task duration, completion of key actions (eg, checking oxygen connections, confirming pulse oximeter clip placement, adjusting oxygen flow rate, assessing consciousness, and checking pulse), and final decision time.

Participants spent an average (SD) of 151.05 (60.94) seconds on the simple case and 132.95 (SD 67.58) seconds on the complex case, showing no significant difference (*t*_69_=1.17, *P*=.11). The average number of tasks completed out of the 6 subtasks was 3.19 in the simple case and 4.33 in the complex case (*t*_69_=−3.02, *P*=.002). Correlation and multiple regression analysis revealed no significant relationships between task duration, number of tasks completed, satisfaction, or confidence predicted posttraining confidence except pretraining confidence (Multimedia Appendix 2).

### Focus Group Interview

Simulation-based education aims not only to enhance performance within controlled scenarios but also to improve the transfer of skills to actual clinical practice. To explore this transfer, FGIs were conducted with the participants at 1 and 5 months after the training. In addition, FGIs were also conducted with the tutors at 1 month after the program to gather feedback on the program’s implementation and operation (Table 4).

**Table 4.** Summary of the focus group interviews on simulation education.

Group and category	Illustrative quotations
Intern	
Advantages of the education program	“It provided a realistic and authentic experience closely resembling what to do in actual clinical situations, such as when a patient’s condition changes suddenly.” (VR^a^ and HFS^b^) “I was really panicked, and it felt like a real situation because the alarms were going off and I felt like I was the only one there.” (VR and HFS) “The detailed and realistic depiction of clinical changes, such as pallor as oxygen saturation changes, were immersive.” (VR) “It was helpful to learn step by step on what to check and do first in an emergency situation.” (VR) “Interactions were only created for the items that needed to be checked, and having this option made learning the procedure fast and efficient.” (VR) “I could repeat the learning as many times as I needed.” (VR) “We were able to learn at our own pace." (VR) “With no fixed answers, the learning felt more like real situations and allowed for greater freedom.” (HFS) “I found the interactive approach to be more helpful than the lectures.” (HFS) “Being able to train with real equipment allowed for experiential learning.” (HFS)
Limitations and areas for improvement	“Because the simulations lacked realistic manipulation, I think additional training with real equipment is necessary.” (VR) “The preset choices limited interaction and reduced the flexibility of the learning process.” (VR) “The system’s responsiveness was a bit low, making it difficult to perform certain tasks.” (VR) “My HMD^c^ device malfunctions caused inconvenience.” (VR) “I think mannequins are limited in their capability to fully depict detailed scenarios.” (HFS) “The high cost and limited availability of equipment made it hard to access when needed.” (HFS) “I couldn’t progress at my own pace during the training.” (HFS) “Not all interns were able to participate directly, so it would be great if there were more opportunities for them to freely join and experience the training. To achieve this, the tutor should minimize direct involvement and focus primarily on a supervising role.” (HFS) “Scenarios with various approaches, where the participant’s actions determine the patient’s recovery, are necessary.” (VR and HFS)
The usefulness of the education program in real clinical situations	<One month after the training> “In real situations where a patient’s oxygen saturation was dropping, I knew what to check and was able to assess the critical factors quickly.” (VR and HFS) “I developed the habit of checking the oxygen tank before patient transport.” (HFS) “While transporting a patient, the patient’s oxygen saturation suddenly dropped, and instead of blindly calling for CPR^d^, I was able to determine that the oxygen tank had run out of oxygen and resolve it by transporting the patient directly to the nearest ward.” (VR) “I felt more confident and less anxious in real situations.” (VR and HFS)
	<Five months after the training> “While transporting a patient with a ventilator, the ventilator alarm kept going off, and when I checked the patient’s condition and the line connection, I found that the ventilator’s line was connected incorrectly, so I fixed the misconnection, and the problem was resolved.” (VR) “When the patient’s oxygen saturation suddenly dropped, a basic check of the line connections and oxygen levels showed no abnormalities, leading me to believe it was an exacerbation of the patient’s underlying condition, and I was able to take action to improve the patient’s condition, first by maximizing the oxygen flow and then by reporting to a supervisor to insert a percutaneous drainage into the lungs.” (VR) “I was transporting a critically ill pediatric patient to the MRI^e^ room when his oxygen levels began to drop. Despite the many connected lines, I noticed the oxygen line was disconnected. After reconnecting it, his condition improved.” (HFS)
Tutor	
Advantages of the education program	“It was an engaging teaching method that resulted in high tutor satisfaction with the overall training experience.” (VR and HFS) “The training allowed the interns to successfully achieve most of their learning objectives, demonstrating the educational effectiveness of these new approaches.” (VR and HFS) "The 2-stage training program, based on difficulty, helped the interns achieve their learning objectives.” (VR and HFS) “Real-time observation of intern performance was possible.” (HFS) “We were able to provide specific and immediate feedback.” (HFS) “I was able to deliver the same training to a large group of interns simultaneously.” (VR) “Following predeveloped educational materials for individual learning made things easier for me as an instructor.” (VR)
Limitations and areas for improvement	“Since tutors were unable to observe each interns’ performance in real time, we had to provide collective feedback to interns at the end of the training.” (VR) “We need a system for real-time monitoring, or automated checklist systems to easily keep an eye on how well the interns are performing.” (VR) “We need a multiplayer platform to make peer learning possible.” (VR) “It was challenging to provide repeated learning and deliver training tailored to the intern’s level.” (HFS) “The current system, which requires tutors to manually change system settings that change as the scenario progresses, needs improvement.” (HFS) “Some of the interns actually participated in the simulated role-plays, while others remained as observers, so we need to make sure that the training is delivered in a way that engages as many interns as possible.” (HFS) “We need dedicated scenarios to fully use the diverse functions of HFS.” (HFS) “The training methods should be adjusted based on the content being taught.” (VR and HFS)

a VR: virtual reality.

b HFS: high-fidelity simulation.

c HMD: head-mounted display.

d CPR: cardiopulmonary resuscitation.

e MRI: magnetic resonance imaging.

### FGI at 1 Month

In total, 5 interns (3 in VR and 2 in HFS) and 4 tutors participated. Interns from both groups described the distinct benefits of their respective training methodologies in preparing them for real-world clinical practice. They also highlighted the value of training in highly controlled yet realistic environments, which appeared to alleviate anxiety and deepen their comprehension of critical desaturation management protocols. VR participants noted the unparalleled immersive and focused nature of the environment, whereas HFS participants appreciated the hands-on realism and flexibility that mirrored real clinical uncertainty. Both approaches were seen as complementary: VR appeared to foster concentration and repeated practice, while HFS seemed to promote situational adaptability.

Regarding the participant’s comment on the efficiency of focused interactions in VR, this reflects the contingent feedback mechanism inherent in both training modalities. In both VR and HFS, the simulation was designed so that when a learner identified a clinical issue and implemented the appropriate intervention, the system provided an immediate physiological reaction, such as stabilization of vital signs or a change in the patient’s status. This equivalence in responsiveness suggests that, regardless of the medium, the learner experiences a consistent “action-reaction” loop.

The tutors expressed overall satisfaction with the training outcomes and process, observing that both the VR and HFS programs effectively supported interns in achieving their learning goals. They praised VR for scalability and standardized materials, though they pointed out the absence of real-time monitoring and immediate, personalized feedback. HFS enabled direct observation and instant feedback but was limited by resource demands and unequal engagement among interns. Tutors suggested the future integration of automated checklists and multiplayer platforms to enhance feedback and encourage peer learning.

### FGI at 5 Months

A follow-up FGI with 4 interns (2 in VR and 2 in HFS) explored how the participants perceived their ability to handle emergencies in real clinical settings, as part of the behavior change evaluation at the third level of the Kirkpatrick model [14]. The interns reported that they were able to effectively manage oxygen desaturation events by systematically assessing oxygen delivery and identifying underlying causes, including complex cases such as ventilator-related desaturation. They reported sustained improvements in their perceived ability to handle desaturation emergencies months after the training (median self-rating scores on a 10-point scale: 0.75 (range 0‐2) before training, 3.25 (range 2‐5) at 1 month, and 7.25 (range 5.5‐8) at 5 month), suggesting potential behavioral transfer of learning to practice.

## Discussion

### Principal Findings

This study compared the educational effectiveness of VR and HFS training in preparing medical interns to manage oxygen desaturation. Both methods significantly improved interns’ self-reported confidence and perceived preparedness, with no substantial difference in overall confidence change between groups. Pretraining preference favored VR education, whereas posttraining satisfaction and perceived authenticity were higher in the HFS group.

Although this study primarily assessed confidence rather than objective competence, confidence in managing acute emergencies can reflect psychological readiness and decision-making under pressure. In high-stress situations such as hypoxia, the ability to apply existing knowledge swiftly often determines performance more than technical skill alone. The focus on oxygen desaturation was intentional, emphasizing cognitive and situational judgment rather than procedural execution. VR simulation aimed to help learners practice rapid assessment and response under stress, strengthening cognitive readiness and composure rather than manual skills. Although increased confidence does not equate to competence, it may reasonably reflect perceived preparedness to act effectively in real clinical contexts.

### Comparison With the Literature

Consistent with prior simulation-based education literature [23-25], the significant increase in self-confidence observed in the VR and HFS training groups underscores the value of simulation-based education. In contrast, satisfaction and perceived authenticity were greater with HFS. Although VR more easily reproduces challenging clinical scenarios [26-28], participants perceived higher realism when using actual medical equipment in HFS. Qualitative feedback provided deeper insights into these findings: interns reported that VR training enhanced their composure and independent decision-making in real situations, as immersive warning sounds and the requirement to manage scenarios alone closely mirrored real-life experiences. This perception may reflect differences in how learners experienced the interaction structure, which was implemented similarly in both VR and HFS but may have been more salient in the VR experience. However, the participants noted that the experience would be enhanced by the ability to physically interact with actual equipment, suggesting opportunities to improve VR realism through mixed or augmented reality and haptic feedback.

### Implications of the Findings

VR education engages learners in an active and central role, but it also brings challenges during debriefing and feedback, key components of simulation-based education. Although the overall structure of debriefing was comparable across groups, learners experienced feedback differently during the simulation. In the HFS group, instructors could directly observe learners’ actions and errors in real time, which may have enabled more specific and context-rich feedback during debriefing, representing one of the major strengths of HFS. In contrast, real-time mirroring was not possible in the VR sessions due to the use of HMDs. Instead, postscenario feedback was provided through structured summaries and predefined prompts, which offered flexibility but may have provided less depth than informed by direct instructor observation in HFS settings. Interestingly, learners did not express dissatisfaction with VR debriefing, suggesting that immersion and autonomy may have compensated for limited instructor feedback. Because both groups received structured debriefing as part of the educational intervention, this shared component may have reduced observable differences between VR and HFS, potentially diluting the treatment effect specific to the simulation modality itself.

Future VR models should consider incorporating hybrid approaches, such as AI-assisted feedback, real-time instructor input via integrated communication tools, or structured peer discussions, to enhance reflective learning and clinical reasoning. Furthermore, it is important to recognize that cultural factors may influence the effectiveness and format of debriefing; learners in high-power distance cultures may hesitate to participate openly unless psychological safety is deliberately structured. As noted in prior studies [29,30], culturally adapted debriefing frameworks could help optimize the educational impact of VR-based simulation across diverse settings. Further research is required to clarify the impact of debriefing methods in VR-based training, as current evidence remains inconclusive [31].

### Limitations of the Study

This study had several limitations. First, the trial was conducted at a single tertiary academic center within a specific institutional and cultural context, and participants were a relatively homogeneous cohort of incoming interns, which limits the generalizability of the findings. Because this study was not designed as a noninferiority or equivalence trial, the absence of statistically significant between-group differences should not be interpreted as evidence of equivalence between VR and HFS. Additionally, practical considerations, such as cost of VR implementation, hardware maintenance, and potential usability challenges in high-pressure clinical environments, may constrain the broader adoption of VR-based training. Multicenter studies across diverse training environments and learner populations will be necessary to validate and extend these findings.

Second, the primary quantitative outcomes focused on self-reported confidence and satisfaction rather than objectively measured performance or long-term retention. Although an objective structured clinical examination was initially planned to evaluate posttraining performance, it was not feasible during the study period [32]. Accordingly, the qualitative findings should be interpreted as perceived learning transfer rather than objectively verified behavior change, and their interpretive value is limited by the small, exploratory sample. While this approach provided insight into learners’ perceived preparedness in relatively straightforward emergency scenarios, it limits conclusions regarding actual performance improvement. Future studies should incorporate objective performance measures, structured observer-rated assessments, longer-term follow-up, and mixed-method designs to more rigorously evaluate learning outcomes and behavioral change over time.

Third, while debriefing occurred after scenario completion in both groups, differences in instructors’ ability to observe learners during the simulation may have influenced the depth of feedback provided during debriefing. Although both modalities shared a similar action–reaction feedback structure, the structured interaction design of the VR environment may have shaped how learners perceived guidance and autonomy, which should be considered when interpreting differences in learner experience. Direct, real-time feedback during the simulation was limited in the VR group, which could be addressed in future studies by integrating mirroring or hybrid feedback methods. Finally, participants’ previous experience with VR was not formally measured; collecting these data would improve the accuracy and interpretation of the results.

### Conclusions

In conclusion, both VR and HFS improved interns’ self-reported confidence and perceived preparedness for managing acute oxygen desaturation. Although satisfaction was higher with HFS, we did not detect a statistically significant between-group difference in confidence change. VR may complement HFS as a scalable educational option in settings where access to mannequin-based simulation is limited. Future work should evaluate objective performance outcomes, longer-term retention, implementation feasibility, and generalizability across diverse institutions and learner groups.

## Supplementary material

10.2196/71455 Multimedia Appendix 1 Baseline characteristics.

10.2196/71455 Multimedia Appendix 2 Correlation of time spent and task resolution scores by case extracted from log data with posttraining confidence change and satisfaction with training.

10.2196/71455 Checklist 1 CONSORT-eHEALTH checklist v1.6.1 and Clinical trial protocol.

## Abbreviations

CONSORT: Consolidated Standards of Reporting Trials
FGI: focus group interview
HFS: high-fidelity simulation
HMD: head-mounted display
UEQ-S: User Experience Questionnaire-Short
VR: virtual reality
