# Supplementary material for: Immersive Virtual Reality Training to Improve Novice Physicians’ Emergency Response Skills: Randomized Controlled Trial
Source: JMIR Med Educ. 2026 Mar 19;12:e71455. doi: 10.2196/71455 (PMC13009707; doi:10.2196/71455)
Supplement: Multimedia Appendix 1 [file mededu-v12-e71455-s001.doc]

**Table S1.**

|  | |  | HFS^a^ (n=85) | VR^b^ (n=79) | *P* value |  |
| --- | --- | --- | --- | --- | --- | --- |
| **Age** | | | 27.2±2.6 | 26.9±1.8 | 0.29 |  |
| **Gender** | Male | | 56 (65.9%) | 46 (58.2%) | 0.34 |  |
|  | | Female | 29 (34.1%) | 33 (41.8%) |  |  |
| **Preference for educational methods** | | Lecture | 9 (12.4%) | 13 (19.7%) | <0.001 |  |
|  | | VR simulation | 19 (26.0%) | 44 (66.7%) |  |  |
|  | | HFS | 45 (61.6%) | 9 (13.6%) |  |  |

^a^HFS: high-fidelity simulation.

^b^VR: virtual reality.
