# Supplementary material for: Immersive Virtual Reality Training to Improve Novice Physicians’ Emergency Response Skills: Randomized Controlled Trial
Source: JMIR Med Educ. 2026 Mar 19;12:e71455. doi: 10.2196/71455 (PMC13009707; doi:10.2196/71455)
Supplement: Multimedia Appendix 2 [file mededu-v12-e71455-s002.doc]

**Table S1.** Correlation of time spent and task resolution scores by case extracted from log data with post-training confidence change and satisfaction with training^a^

| **Variables** | **Log data** | | | **Pre-training** | | | | | **Post-training** | | | | | | | | | |
| --- | --- | --- | --- | --- | --- | --- | --- | --- | --- | --- | --- | --- | --- | --- | --- | --- | --- | --- |
|  | **S_Solved Tasks** | **C_Total Time** | **C_Solved Tasks** | **Confidence_1^f^** | **Confidence_2^g^** | **Confidence_3^h^** | **Confidence_4^i^** | **Confidence_5^j^** | **Confidence_1** | **Confidence_2** | **Confidence_3** | **Confidence_4** | **Confidence_5** | **Satisfaction_1^k^** | **Satisfaction_2^l^** | **Satisfaction_3^m^** | **Satisfaction_4^n^** |  |
| **S_Total Time^b^** | 0.521  ** | 0.125 | 0.319 | 0.030 | -0.028 | 0.012 | -0.031 | 0.022 | -0.153 | -0.353 | -0.286 | -0.362  * | -0.285 | -0.205 | -0.134 | -0.068 | 0.031 |  |
| **S_Solved Tasks^c^** | 1 | -0.061 | 0.178 | 0.137 | 0.150 | -0.015 | 0.007 | 0.033 | 0.121 | -0.004 | -0.041 | 0.012 | -0.024 | -0.464  ** | -0.286 | -0.255 | -0.327 |  |
| **C_Total Time^d^** |  | 1 | 0.325  * | -0.181 | -0.066 | -0.057 | -0.009 | -0.120 | -0.159 | -0.209 | -0.067 | -0.088 | -0.123 | 0.063 | -0.018 | 0.134 | 0.104 |  |
| **C_Solved Tasks^e^** |  |  | 1 | -0.225 | -0.118 | -0.207 | -0.089 | -0.201 | -0.156 | -0.088 | -0.130 | -0.161 | -0.167 | 0.097 | 0.183 | 0.136 | 0.055 |  |

^a^ Values are Pearson correlation coefficient. **P* < .05, ***P* < .01 indicate statistical significance.

**^b^**S_Total Time: Time spent for simple case.

**^c^**S_Solved Tasks: The sum of the number of clear tasks in the simple case.

**^d^**C_Total Time: Time spent for complex case.

^e^C_Solved Tasks: The sum of the number of clear tasks in the complex case.

**^f^**Confidence_1: Confidence in managing patients with seizures.

**^g^**Confidence_2: Confidence in managing patients with desaturation.

^h^Confidence_3: Confidence in managing patients with anaphylaxis.

^i^Confidence_4: Confidence in general patient care abilities.

**^j^**Confidence_5: Confidence in general clinical skill competency.

**^k^**Satisfaction_1: Overall satisfaction with training.

**^l^**Satisfaction_2: Satisfaction with length of course.

**^m^**Satisfaction_3: Satisfaction with tutor’s guidance.

**^n^**Satisfaction_4: Satisfaction with the realism of the training.
