# Supplementary material for: Immersive Virtual Reality Training to Improve Novice Physicians’ Emergency Response Skills: Randomized Controlled Trial
Source: JMIR Med Educ. 2026 Mar 19;12:e71455. doi: 10.2196/71455 (PMC13009707; doi:10.2196/71455)
Supplement: Checklist 1 [file mededu-v12-e71455-s003.pdf]

CONSORT-EHEALTH (V 1.6.1) - Submission/Publication Form

The CONSORT-EHEALTH checklist is intended for authors of randomized trials evaluating web-based and Internet-based applications/interventions, including mobile interventions, electronic games (incl multiplayer games), social media, certain telehealth applications, and other interactive and/or networked electronic applications. Some of the items (e.g. all subitems under item 5 - description of the intervention) may also be applicable for other study designs.

The goal of the CONSORT EHEALTH checklist and guideline is to be
a) a guide for reporting for authors of RCTs,
b) to form a basis for appraisal of an ehealth trial (in terms of validity)

CONSORT-EHEALTH items/subitems are MANDATORY reporting items for studies published in the Journal of Medical Internet Research and other journals / scienti c societies endorsing the checklist.

Items numbered 1., 2., 3., 4a., 4b etc are original CONSORT or CONSORT-NPT (non-pharmacologic treatment) items.
Items with Roman numerals (i., ii, iii, iv etc.) are CONSORT-EHEALTH extensions/clari cations.

As the CONSORT-EHEALTH checklist is still considered in a formative stage, we would ask that you also RATE ON A SCALE OF 1-5 how important/useful you feel each item is FOR THE PURPOSE OF THE CHECKLIST and reporting guideline (optional).

Mandatory reporting items are marked with a red *.
In the textboxes, either copy & paste the relevant sections from your manuscript into this form - please include any quotes from your manuscript in QUOTATION MARKS,
or answer directly by providing additional information not in the manuscript, or elaborating on why the item was not relevant for this study.

YOUR ANSWERS WILL BE PUBLISHED AS A SUPPLEMENTARY FILE TO YOUR PUBLICATION IN JMIR AND ARE CONSIDERED PART OF YOUR PUBLICATION (IF ACCEPTED).
Please ll in these questions diligently. Information will not be copyedited, so please use proper spelling and grammar, use correct capitalization, and avoid abbreviations.

DO NOT FORGET TO SAVE AS PDF _AND_ CLICK THE SUBMIT BUTTON SO YOUR ANSWERS ARE IN OUR DATABASE !!!

Citation Suggestion (if you append the pdf as Appendix we suggest to cite this paper in the caption):
Eysenbach G, CONSORT-EHEALTH Group
CONSORT-EHEALTH: Improving and Standardizing Evaluation Reports of Web-based and Mobile Health Interventions
J Med Internet Res 2011;13(4):e126

URL: http://www.jmir.org/2011/4/e126/
doi: 10.2196/jmir.1923
PMID: 22209829

yeonjuhuh.md@gmail.com 계정 전환

비공개

초안 저장됨

* 표시는 필수 질문임

Your name *

First Last

Yeon-Ju Huh

Primary Affiliation (short), City, Country *

University of Toronto, Toronto, Canada

Seoul National University College of Medicine,

Your e-mail address *

abc@gmail.com

youhear12@snu.ac.kr

Title of your manuscript *

Provide the (draft) title of your manuscript.

Immersive Virtual Reality Training to Improve Novice Physicians' Emergency Response Skills: A Randomized Controlled Trial

Name of your App/Software/Intervention *

If there is a short and a long/alternate name, write the short name rst and add the long name in brackets.

Unity Technologies platform/ Virtual Reality vs

Evaluated Version (if any)

e.g. "V1", "Release 2017-03-01", "Version 2.0.27913"

내 답변

Language(s) *

What language is the intervention/app in? If multiple languages are available, separate by comma (e.g. "English, French")

English

URL of your Intervention Website or App

e.g. a direct link to the mobile app on app in appstore (itunes, Google Play), or URL of the website. If the intervention is a DVD or hardware, you can also link to an Amazon page.

내 답변

URL of an image/screenshot (optional)

내 답변

Accessibility *

Can an enduser access the intervention presently?

- ○ access is free and open
- ◉ access only for special usergroups, not open
- ○ access is open to everyone, but requires payment/subscription/in-app purchases
- ○ app/intervention no longer accessible
- ○ 기타:

Primary Medical Indication/Disease/Condition *

e.g. "Stress", "Diabetes", or de ne the target group in brackets after the condition, e.g. "Autism (Parents of children with)", "Alzheimers (Informal Caregivers of)"

Emergency situation/Hypoxia/Novice doctor

Primary Outcomes measured in trial *

comma-separated list of primary outcomes reported in the trial

- To evaluate the improvement in confidenc

Secondary/other outcomes

Are there any other outcomes the intervention is expected to affect?

- To assess the usability of the VR and HFS systems.
- To explore real-world applicability and behavioral changes through focus group interviews (FGIs).

Recommended "Dose" *

What do the instructions for users say on how often the app should be used?

- ( ) Approximately Daily
- ( ) Approximately Weekly
- ( ) Approximately Monthly
- ( ) Approximately Yearly
- (•) "as needed"
- ( ) 기타:

Approx. Percentage of Users (starters) still using the app as recommended after 3 months *

- (•) unknown / not evaluated
- ( ) 0-10%
- ( ) 11-20%
- ( ) 21-30%
- ( ) 31-40%
- ( ) 41-50%
- ( ) 51-60%
- ( ) 61-70%
- ( ) 71%-80%
- ( ) 81-90%
- ( ) 91-100%
- ( ) 기타:

Overall, was the app/intervention effective? *

- ○ yes: all primary outcomes were signi cantly better in intervention group vs control
- ◉ partly: SOME primary outcomes were signi cantly better in intervention group vs control
- ○ no statistically signi cant difference between control and intervention
- ○ potentially harmful: control was signi cantly better than intervention in one or more outcomes
- ○ inconclusive: more research is needed
- ○ 기타:

Article Preparation Status/Stage *

At which stage in your article preparation are you currently (at the time you ll in this form)

- ○ not submitted yet - in early draft status
- ◉ not submitted yet - in late draft status, just before submission
- ○ submitted to a journal but not reviewed yet
- ○ submitted to a journal and after receiving initial reviewer comments
- ○ submitted to a journal and accepted, but not published yet
- ○ published
- ○ 기타:

Journal *

If you already know where you will submit this paper (or if it is already submitted), please provide the journal name (if it is not JMIR, provide the journal name under "other")

- ○ not submitted yet / unclear where I will submit this
- ◉ Journal of Medical Internet Research (JMIR)
- ○ JMIR mHealth and UHealth
- ○ JMIR Serious Games
- ○ JMIR Mental Health
- ○ JMIR Public Health
- ○ JMIR Formative Research
- ○ Other JMIR sister journal
- ○ 기타:

Is this a full powered effectiveness trial or a pilot/feasibility trial? *

- ○ Pilot/feasibility
- ◉ Fully powered

Manuscript tracking number *

If this is a JMIR submission, please provide the manuscript tracking number under "other" (The ms tracking number can be found in the submission acknowledgement email, or when you login as author in JMIR. If the paper is already published in JMIR, then the ms tracking number is the four-digit number at the end of the DOI, to be found at the bottom of each published article in JMIR)

- ◉ no ms number (yet) / not (yet) submitted to / published in JMIR
- ○ 기타:

TITLE AND ABSTRACT

1a) TITLE: Identification as a randomized trial in the title

1a) Does your paper address CONSORT item 1a? *

I.e does the title contain the phrase "Randomized Controlled Trial"? (if not, explain the reason under "other")

- (●) yes
- ( ) 기타:

1a-i) Identify the mode of delivery in the title

Identify the mode of delivery. Preferably use “web-based” and/or “mobile” and/or “electronic game” in the title. Avoid ambiguous terms like “online”, “virtual”, “interactive”. Use “Internet-based” only if Intervention includes non-web-based Internet components (e.g. email), use “computer-based” or “electronic” only if o ine products are used. Use “virtual” only in the context of “virtual reality” (3-D worlds). Use “online” only in the context of “online support groups”. Complement or substitute product names with broader terms for the class of products (such as “mobile” or “smart phone” instead of “iphone”), especially if the application runs on different platforms.

	1	2	3	4	5	
subitem not at all important	( )	( )	( )	( )	(●)	essential

선택해제

Does your paper address subitem 1a-i? *

Copy and paste relevant sections from manuscript title (include quotes in quotation marks "like this" to indicate direct quotes from your manuscript), or elaborate on this item by providing additional information not in the ms, or brie y explain why the item is not applicable/relevant for your study

"Immersive Virtual Reality"

1a-ii) Non-web-based components or important co-interventions in title

Mention non-web-based components or important co-interventions in title, if any (e.g., “with telephone support”).

	1	2	3	4	5	
subitem not at all important	◉	○	○	○	○	essential

선택해제

Does your paper address subitem 1a-ii?

Copy and paste relevant sections from manuscript title (include quotes in quotation marks "like this" to indicate direct quotes from your manuscript), or elaborate on this item by providing additional information not in the ms, or brie y explain why the item is not applicable/relevant for your study

내 답변

1a-iii) Primary condition or target group in the title

Mention primary condition or target group in the title, if any (e.g., “for children with Type I Diabetes”) Example: A Web-based and Mobile Intervention with Telephone Support for Children with Type I Diabetes: Randomized Controlled Trial

	1	2	3	4	5	
subitem not at all important	○	○	○	○	◉	essential

선택해제

Does your paper address subitem 1a-iii? *

Copy and paste relevant sections from manuscript title (include quotes in quotation marks "like this" to indicate direct quotes from your manuscript), or elaborate on this item by providing additional information not in the ms, or brie y explain why the item is not applicable/relevant for your study

"Training to Improve Novice Physicians’ Emergency Response Skills"

1b) ABSTRACT: Structured summary of trial design, methods, results, and conclusions

NPT extension: Description of experimental treatment, comparator, care providers, centers, and blinding status.

1b-i) Key features/functionalities/components of the intervention and comparator in the METHODS section of the ABSTRACT

Mention key features/functionalities/components of the intervention and comparator in the abstract. If possible, also mention theories and principles used for designing the site. Keep in mind the needs of systematic reviewers and indexers by including important synonyms. (Note: Only report in the abstract what the main paper is reporting. If this information is missing from the main body of text, consider adding it)

	1	2	3	4	5	
subitem not at all important	○	○	○	○	○	essential

Does your paper address subitem 1b-i? *

Copy and paste relevant sections from the manuscript abstract (include quotes in quotation marks "like this" to indicate direct quotes from your manuscript), or elaborate on this item by providing additional information not in the ms, or brie y explain why the item is not applicable/relevant for your study

"A randomized controlled trial was conducted with 168 medical interns assigned to VR (n = 81) or HFS (n = 87) training simulated managing patients with low oxygen saturation."

1b-ii) Level of human involvement in the METHODS section of the ABSTRACT

Clarify the level of human involvement in the abstract, e.g., use phrases like “fully automated” vs. “therapist/nurse/care provider/physician-assisted” (mention number and expertise of providers involved, if any). (Note: Only report in the abstract what the main paper is reporting. If this information is missing from the main body of text, consider adding it)

	1	2	3	4	5	
subitem not at all important	○	○	○	○	○	essential

Does your paper address subitem 1b-ii?

Copy and paste relevant sections from the manuscript abstract (include quotes in quotation marks "like this" to indicate direct quotes from your manuscript), or elaborate on this item by providing additional information not in the ms, or brie y explain why the item is not applicable/relevant for your study

내 답변

1b-iii) Open vs. closed, web-based (self-assessment) vs. face-to-face assessments in the METHODS section of the ABSTRACT

Mention how participants were recruited (online vs. o ine), e.g., from an open access website or from a clinic or a closed online user group (closed usergroup trial), and clarify if this was a purely web-based trial, or there were face-to-face components (as part of the intervention or for assessment). Clearly say if outcomes were self-assessed through questionnaires (as common in web-based trials). Note: In traditional o ine trials, an open trial (open-label trial) is a type of clinical trial in which both the researchers and participants know which treatment is being administered. To avoid confusion, use “blinded” or “unblinded” to indicated the level of blinding instead of “open”, as “open” in web-based trials usually refers to “open access” (i.e. participants can self-enrol). (Note: Only report in the abstract what the main paper is reporting. If this information is missing from the main body of text, consider adding it)

	1	2	3	4	5	
subitem not at all important	○	○	○	○	○	essential

Does your paper address subitem 1b-iii?

Copy and paste relevant sections from the manuscript abstract (include quotes in quotation marks "like this" to indicate direct quotes from your manuscript), or elaborate on this item by providing additional information not in the ms, or brie y explain why the item is not applicable/relevant for your study

내 답변

1b-iv) RESULTS section in abstract must contain use data

Report number of participants enrolled/assessed in each group, the use/uptake of the intervention (e.g., attrition/adherence metrics, use over time, number of logins etc.), in addition to primary/secondary outcomes. (Note: Only report in the abstract what the main paper is reporting. If this information is missing from the main body of text, consider adding it)

	1	2	3	4	5	
subitem not at all important	○	○	○	○	○	essential

Does your paper address subitem 1b-iv?

Copy and paste relevant sections from the manuscript abstract (include quotes in quotation marks "like this" to indicate direct quotes from your manuscript), or elaborate on this item by providing additional information not in the ms, or brie y explain why the item is not applicable/relevant for your study

내 답변

1b-v) CONCLUSIONS/DISCUSSION in abstract for negative trials

Conclusions/Discussions in abstract for negative trials: Discuss the primary outcome - if the trial is negative (primary outcome not changed), and the intervention was not used, discuss whether negative results are attributable to lack of uptake and discuss reasons. (Note: Only report in the abstract what the main paper is reporting. If this information is missing from the main body of text, consider adding it)

	1	2	3	4	5	
subitem not at all important	○	○	○	○	○	essential

Does your paper address subitem 1b-v?

Copy and paste relevant sections from the manuscript abstract (include quotes in quotation marks "like this" to indicate direct quotes from your manuscript), or elaborate on this item by providing additional information not in the ms, or brie y explain why the item is not applicable/relevant for your study

내 답변

INTRODUCTION

2a) In INTRODUCTION: Scientific background and explanation of rationale

2a-i) Problem and the type of system/solution

Describe the problem and the type of system/solution that is object of the study: intended as stand-alone intervention vs. incorporated in broader health care program? Intended for a particular patient population? Goals of the intervention, e.g., being more cost-effective to other interventions, replace or complement other solutions? (Note: Details about the intervention are provided in “Methods” under 5)

	1	2	3	4	5	
subitem not at all important	○	○	○	○	○	essential

Does your paper address subitem 2a-i? *

Copy and paste relevant sections from the manuscript (include quotes in quotation marks "like this" to indicate direct quotes from your manuscript), or elaborate on this item by providing additional information not in the ms, or brie y explain why the item is not applicable/relevant for your study

"Ensuring that interns and new practitioners are adequately prepared for these high-stakes scenarios is essential for improving patient outcomes and reducing medical errors. A rapid decline in oxygen saturation represents one of the most critical and life-threatening challenges in medical practice, demanding immediate intervention to avert severe complications or death."

2a-ii) Scientific background, rationale: What is known about the (type of) system

Scienti c background, rationale: What is known about the (type of) system that is the object of the study (be sure to discuss the use of similar systems for other conditions/diagnoses, if appropiate), motivation for the study, i.e. what are the reasons for and what is the context for this speci c study, from which stakeholder viewpoint is the study performed, potential impact of ndings [2]. Brie y justify the choice of the comparator.

	1	2	3	4	5	
subitem not at all important	○	○	○	○	○	essential

Does your paper address subitem 2a-ii? *

Copy and paste relevant sections from the manuscript (include quotes in quotation marks "like this" to indicate direct quotes from your manuscript), or elaborate on this item by providing additional information not in the ms, or brie y explain why the item is not applicable/relevant for your study

"Traditional educational approaches, while foundational, often lack the immersive, hands-on experience necessary to equip learners fully for real-world clinical challenges........Simulation-based education has emerged as a vital tool, providing a safe and controlled environment for developing and practicing skills."

2b) In INTRODUCTION: Specific objectives or hypotheses

Does your paper address CONSORT subitem 2b? *

Copy and paste relevant sections from the manuscript (include quotes in quotation marks "like this" to indicate direct quotes from your manuscript), or elaborate on this item by providing additional information not in the ms, or brie y explain why the item is not applicable/relevant for your study

"This study aimed to address this gap by evaluating and comparing the educational outcomes of VR and HFS in preparing interns to manage emergencies."

METHODS

3a) Description of trial design (such as parallel, factorial) including allocation ratio

Does your paper address CONSORT subitem 3a? *

Copy and paste relevant sections from the manuscript (include quotes in quotation marks "like this" to indicate direct quotes from your manuscript), or elaborate on this item by providing additional information not in the ms, or brie y explain why the item is not applicable/relevant for your study

"A two-arm parallel randomized controlled trial (RCT) was conducted to compare the effectiveness of VR and HFS educational methods........ Participants were randomly allocated to either the VR or HFS training group using simple randomization."

3b) Important changes to methods after trial commencement (such as eligibility criteria), with reasons

Does your paper address CONSORT subitem 3b? *

Copy and paste relevant sections from the manuscript (include quotes in quotation marks "like this" to indicate direct quotes from your manuscript), or elaborate on this item by providing additional information not in the ms, or brie y explain why the item is not applicable/relevant for your study

There were no changes to the methods, including eligibility criteria or trial procedures, after the commencement of the trial.

3b-i) Bug fixes, Downtimes, Content Changes

Bug xes, Downtimes, Content Changes: ehealth systems are often dynamic systems. A description of changes to methods therefore also includes important changes made on the intervention or comparator during the trial (e.g., major bug xes or changes in the functionality or content) (5-iii) and other “unexpected events” that may have in uenced study design such as staff changes, system failures/downtimes, etc. [2].

	1	2	3	4	5	
subitem not at all important	○	○	○	○	○	essential

Does your paper address subitem 3b-i?

Copy and paste relevant sections from the manuscript (include quotes in quotation marks "like this" to indicate direct quotes from your manuscript), or elaborate on this item by providing additional information not in the ms, or brie y explain why the item is not applicable/relevant for your study

내 답변

4a) Eligibility criteria for participants

Does your paper address CONSORT subitem 4a? *

Copy and paste relevant sections from the manuscript (include quotes in quotation marks "like this" to indicate direct quotes from your manuscript), or elaborate on this item by providing additional information not in the ms, or brie y explain why the item is not applicable/relevant for your study

"168 prospective interns who had recently graduated from medical school and were about to begin their internships were randomly divided into two groups for the RCT"

4a-i) Computer / Internet literacy

Computer / Internet literacy is often an implicit “de facto” eligibility criterion - this should be explicitly clari ed.

	1	2	3	4	5	
subitem not at all important	○	○	○	○	○	essential

Does your paper address subitem 4a-i?

Copy and paste relevant sections from the manuscript (include quotes in quotation marks "like this" to indicate direct quotes from your manuscript), or elaborate on this item by providing additional information not in the ms, or brie y explain why the item is not applicable/relevant for your study

내 답변

4a-ii) Open vs. closed, web-based vs. face-to-face assessments:

Open vs. closed, web-based vs. face-to-face assessments: Mention how participants were recruited (online vs. o ine), e.g., from an open access website or from a clinic, and clarify if this was a purely web-based trial, or there were face-to-face components (as part of the intervention or for assessment), i.e., to what degree got the study team to know the participant. In online-only trials, clarify if participants were quasi-anonymous and whether having multiple identities was possible or whether technical or logistical measures (e.g., cookies, email con rmation, phone calls) were used to detect/prevent these.

	1	2	3	4	5	
subitem not at all important	○	○	○	○	○	essential

Does your paper address subitem 4a-ii? *

Copy and paste relevant sections from the manuscript (include quotes in quotation marks "like this" to indicate direct quotes from your manuscript), or elaborate on this item by providing additional information not in the ms, or brie y explain why the item is not applicable/relevant for your study

1. Recruitment Method:
- Offline recruitment: Participants were recruited from the graduating class of a medical school.
- No public websites or online recruitment methods were used.

2. Face-to-Face Components:
- All assessments and interventions were conducted face-to-face.
- Participants completed training and surveys at a designated simulation center.

4a-iii) Information giving during recruitment

Information given during recruitment. Specify how participants were briefed for recruitment and in the informed consent procedures (e.g., publish the informed consent documentation as appendix, see also item X26), as this information may have an effect on user self-selection, user expectation and may also bias results.

	1	2	3	4	5	
subitem not at all important	○	○	○	○	○	essential

Does your paper address subitem 4a-iii?

Copy and paste relevant sections from the manuscript (include quotes in quotation marks "like this" to indicate direct quotes from your manuscript), or elaborate on this item by providing additional information not in the ms, or brie y explain why the item is not applicable/relevant for your study

내 답변

4b) Settings and locations where the data were collected

Does your paper address CONSORT subitem 4b? *

Copy and paste relevant sections from the manuscript (include quotes in quotation marks "like this" to indicate direct quotes from your manuscript), or elaborate on this item by providing additional information not in the ms, or brie y explain why the item is not applicable/relevant for your study

"After obtaining informed consent, the participants were assigned identification numbers and randomly allocated to either the VR or HFS training group using simple randomization. ... This study received approval from the Seoul National University Hospital Institutional Review Board"

4b-i) Report if outcomes were (self-)assessed through online questionnaires

Clearly report if outcomes were (self-)assessed through online questionnaires (as common in web-based trials) or otherwise.

	1	2	3	4	5	
subitem not at all important	○	○	○	○	○	essential

Does your paper address subitem 4b-i? *

Copy and paste relevant sections from the manuscript (include quotes in quotation marks "like this" to indicate direct quotes from your manuscript), or elaborate on this item by providing additional information not in the ms, or brie y explain why the item is not applicable/relevant for your study

The training was conducted face-to-face, and the surveys were also administered in person before and after the training sessions.

4b-ii) Report how institutional affiliations are displayed

Report how institutional a liations are displayed to potential participants [on ehealth media], as a liations with prestigious hospitals or universities may affect volunteer rates, use, and reactions with regards to an intervention.(Not a required item – describe only if this may bias results)

	1	2	3	4	5	
subitem not at all important	○	○	○	○	○	essential

Does your paper address subitem 4b-ii?

Copy and paste relevant sections from the manuscript (include quotes in quotation marks "like this" to indicate direct quotes from your manuscript), or elaborate on this item by providing additional information not in the ms, or brie y explain why the item is not applicable/relevant for your study

내 답변

5) The interventions for each group with sufficient details to allow replication, including how and when they were actually administered

5-i) Mention names, credential, affiliations of the developers, sponsors, and owners

Mention names, credential, a liations of the developers, sponsors, and owners [6] (if authors/evaluators are owners or developer of the software, this needs to be declared in a “Con ict of interest” section or mentioned elsewhere in the manuscript).

	1	2	3	4	5	
subitem not at all important	○	○	○	○	○	essential

Does your paper address subitem 5-i?

Copy and paste relevant sections from the manuscript (include quotes in quotation marks "like this" to indicate direct quotes from your manuscript), or elaborate on this item by providing additional information not in the ms, or brie y explain why the item is not applicable/relevant for your study

내 답변

5-ii) Describe the history/development process

Describe the history/development process of the application and previous formative evaluations (e.g., focus groups, usability testing), as these will have an impact on adoption/use rates and help with interpreting results.

	1	2	3	4	5	
subitem not at all important	○	○	○	○	○	essential

Does your paper address subitem 5-ii?

Copy and paste relevant sections from the manuscript (include quotes in quotation marks "like this" to indicate direct quotes from your manuscript), or elaborate on this item by providing additional information not in the ms, or brie y explain why the item is not applicable/relevant for your study

내 답변

5-iii) Revisions and updating

Revisions and updating. Clearly mention the date and/or version number of the application/intervention (and comparator, if applicable) evaluated, or describe whether the intervention underwent major changes during the evaluation process, or whether the development and/or content was “frozen” during the trial. Describe dynamic components such as news feeds or changing content which may have an impact on the replicability of the intervention (for unexpected events see item 3b).

	1	2	3	4	5	
subitem not at all important	○	○	○	○	○	essential

Does your paper address subitem 5-iii?

Copy and paste relevant sections from the manuscript (include quotes in quotation marks "like this" to indicate direct quotes from your manuscript), or elaborate on this item by providing additional information not in the ms, or brie y explain why the item is not applicable/relevant for your study

내 답변

5-iv) Quality assurance methods

Provide information on quality assurance methods to ensure accuracy and quality of information provided [1], if applicable.

	1	2	3	4	5	
subitem not at all important	○	○	○	○	○	essential

Does your paper address subitem 5-iv?

Copy and paste relevant sections from the manuscript (include quotes in quotation marks "like this" to indicate direct quotes from your manuscript), or elaborate on this item by providing additional information not in the ms, or brie y explain why the item is not applicable/relevant for your study

내 답변

5-v) Ensure replicability by publishing the source code, and/or providing screenshots/screen-capture video, and/or providing flowcharts of the algorithms used

Ensure replicability by publishing the source code, and/or providing screenshots/screen-capture video, and/or providing owcharts of the algorithms used. Replicability (i.e., other researchers should in principle be able to replicate the study) is a hallmark of scienti c reporting.

	1	2	3	4	5	
subitem not at all important	○	○	○	○	○	essential

Does your paper address subitem 5-v?

Copy and paste relevant sections from the manuscript (include quotes in quotation marks "like this" to indicate direct quotes from your manuscript), or elaborate on this item by providing additional information not in the ms, or brie y explain why the item is not applicable/relevant for your study

내 답변

5-vi) Digital preservation

Digital preservation: Provide the URL of the application, but as the intervention is likely to change or disappear over the course of the years; also make sure the intervention is archived (Internet Archive, webcitation.org, and/or publishing the source code or screenshots/videos alongside the article). As pages behind login screens cannot be archived, consider creating demo pages which are accessible without login.

	1	2	3	4	5	
subitem not at all important	○	○	○	○	○	essential

Does your paper address subitem 5-vi?

Copy and paste relevant sections from the manuscript (include quotes in quotation marks "like this" to indicate direct quotes from your manuscript), or elaborate on this item by providing additional information not in the ms, or brie y explain why the item is not applicable/relevant for your study

내 답변

5-vii) Access

Access: Describe how participants accessed the application, in what setting/context, if they had to pay (or were paid) or not, whether they had to be a member of speci c group. If known, describe how participants obtained “access to the platform and Internet” [1]. To ensure access for editors/reviewers/readers, consider to provide a “backdoor” login account or demo mode for reviewers/readers to explore the application (also important for archiving purposes, see vi).

	1	2	3	4	5	
subitem not at all important	○	○	○	○	○	essential

Does your paper address subitem 5-vii? *

Copy and paste relevant sections from the manuscript (include quotes in quotation marks "like this" to indicate direct quotes from your manuscript), or elaborate on this item by providing additional information not in the ms, or brie y explain why the item is not applicable/relevant for your study

Participants accessed the application in a designated simulation center, where all necessary equipment, including VR devices, was provided by the research team. The participants did not need to pay for access to the application, nor were they compensated for participation. All participants were medical interns from the same hospital, thus forming a specific group. Since this study did not involve a publicly accessible platform, however, a demo video can be provided upon request from editors or reviewers.

5-viii) Mode of delivery, features/functionalities/components of the intervention and comparator, and the theoretical framework

Describe mode of delivery, features/functionalities/components of the intervention and comparator, and the theoretical framework [6] used to design them (instructional strategy [1], behaviour change techniques, persuasive features, etc., see e.g., [7, 8] for terminology). This includes an in-depth description of the content (including where it is coming from and who developed it) [1],” whether [and how] it is tailored to individual circumstances and allows users to track their progress and receive feedback” [6]. This also includes a description of communication delivery channels and – if computer-mediated communication is a component – whether communication was synchronous or asynchronous [6]. It also includes information on presentation strategies [1], including page design principles, average amount of text on pages, presence of hyperlinks to other resources, etc. [1].

	1	2	3	4	5	
subitem not at all important	○	○	○	○	○	essential

Does your paper address subitem 5-viii? *

Copy and paste relevant sections from the manuscript (include quotes in quotation marks "like this" to indicate direct quotes from your manuscript), or elaborate on this item by providing additional information not in the ms, or brie y explain why the item is not applicable/relevant for your study

"168 prospective interns who had recently graduated from medical school and were about to begin their internships were randomly divided into two groups for the RCT (Figure 1). ... Participants simulated scenarios involving desaturation based on their previous knowledge using the specific training method for their group, followed by a debriefing session to reinforce learning. After completing the simulation, the experimental and control groups were debriefed to discuss their performance and enhance their response capabilities (Figure 2)."

5-ix) Describe use parameters

Describe use parameters (e.g., intended “doses” and optimal timing for use). Clarify what instructions or recommendations were given to the user, e.g., regarding timing, frequency, heaviness of use, if any, or was the intervention used ad libitum.

	1	2	3	4	5	
subitem not at all important	○	○	○	○	○	essential

Does your paper address subitem 5-ix?

Copy and paste relevant sections from the manuscript (include quotes in quotation marks "like this" to indicate direct quotes from your manuscript), or elaborate on this item by providing additional information not in the ms, or brie y explain why the item is not applicable/relevant for your study

내 답변

5-x) Clarify the level of human involvement

Clarify the level of human involvement (care providers or health professionals, also technical assistance) in the e-intervention or as co-intervention (detail number and expertise of professionals involved, if any, as well as “type of assistance offered, the timing and frequency of the support, how it is initiated, and the medium by which the assistance is delivered”. It may be necessary to distinguish between the level of human involvement required for the trial, and the level of human involvement required for a routine application outside of a RCT setting (discuss under item 21 – generalizability).

	1	2	3	4	5	
subitem not at all important	○	○	○	○	○	essential

Does your paper address subitem 5-x?

Copy and paste relevant sections from the manuscript (include quotes in quotation marks "like this" to indicate direct quotes from your manuscript), or elaborate on this item by providing additional information not in the ms, or brie y explain why the item is not applicable/relevant for your study

내 답변

5-xi) Report any prompts/reminders used

Report any prompts/reminders used: Clarify if there were prompts (letters, emails, phone calls, SMS) to use the application, what triggered them, frequency etc. It may be necessary to distinguish between the level of prompts/reminders required for the trial, and the level of prompts/reminders for a routine application outside of a RCT setting (discuss under item 21 – generalizability).

	1	2	3	4	5	
subitem not at all important	○	○	○	○	○	essential

Does your paper address subitem 5-xi? *

Copy and paste relevant sections from the manuscript (include quotes in quotation marks "like this" to indicate direct quotes from your manuscript), or elaborate on this item by providing additional information not in the ms, or brie y explain why the item is not applicable/relevant for your study

The face-to-face evaluation was mandatory for the involved participants.

5-xii) Describe any co-interventions (incl. training/support)

Describe any co-interventions (incl. training/support): Clearly state any interventions that are provided in addition to the targeted eHealth intervention, as ehealth intervention may not be designed as stand-alone intervention. This includes training sessions and support [1]. It may be necessary to distinguish between the level of training required for the trial, and the level of training for a routine application outside of a RCT setting (discuss under item 21 – generalizability.

	1	2	3	4	5	
subitem not at all important	○	○	○	○	○	essential

Does your paper address subitem 5-xii? *

Copy and paste relevant sections from the manuscript (include quotes in quotation marks "like this" to indicate direct quotes from your manuscript), or elaborate on this item by providing additional information not in the ms, or brie y explain why the item is not applicable/relevant for your study

When providing the consent form, all participants were given a common explanation about the study. Since most participants had no prior experience with VR, a brief 5-minute tutorial was provided before the training to help them become familiar with the environment.

6a) Completely defined pre-specified primary and secondary outcome measures, including how and when they were assessed

Does your paper address CONSORT subitem 6a? *

Copy and paste relevant sections from the manuscript (include quotes in quotation marks "like this" to indicate direct quotes from your manuscript), or elaborate on this item by providing additional information not in the ms, or brie y explain why the item is not applicable/relevant for your study

"Surveys were conducted in both groups to assess the program, including its educational effectiveness. The survey evaluated overall satisfaction with the educational program, including satisfaction with the training duration, tutor guidance, and perceived authenticity of the educational experience, using a 7-point Likert scale [14]. Educational effectiveness was measured by having participants rate their confidence in handling various scenarios on a 10-point scale [15] before and after the program. In addition to the primary focus on oxygen desaturation management, control questions were included to assess changes in confidence when managing situations, such as seizures or anaphylaxis, which were not directly incorporated into the training. We assessed usability with the UEQ-S [16], along with any experiences of discomfort or sickness associated with VR use, using a 7-point scale. To further evaluate the effect of the program, FGIs were conducted with the interns and tutors 1 and 5 months post-training. These interviews aimed to determine how well the training supported participants in actual patient care during their medical internships, gather feedback on the simulation program, assess educational effectiveness, and identify areas for improvement."

6a-i) Online questionnaires: describe if they were validated for online use and apply CHERRIES items to describe how the questionnaires were designed/deployed

If outcomes were obtained through online questionnaires, describe if they were validated for online use and apply CHERRIES items to describe how the questionnaires were designed/deployed [9].

	1	2	3	4	5	
subitem not at all important	○	○	○	○	○	essential

Does your paper address subitem 6a-i?

Copy and paste relevant sections from manuscript text

내 답변

6a-ii) Describe whether and how "use" (including intensity of use/dosage) was defined/measured/monitored

Describe whether and how "use" (including intensity of use/dosage) was de ned/measured/monitored (logins, log le analysis, etc.). Use/adoption metrics are important process outcomes that should be reported in any ehealth trial.

	1	2	3	4	5	
subitem not at all important	○	○	○	○	○	essential

Does your paper address subitem 6a-ii?

Copy and paste relevant sections from manuscript text

내 답변

6a-iii) Describe whether, how, and when qualitative feedback from participants was obtained

Describe whether, how, and when qualitative feedback from participants was obtained (e.g., through emails, feedback forms, interviews, focus groups).

	1	2	3	4	5	
subitem not at all important	○	○	○	○	○	essential

Does your paper address subitem 6a-iii?

Copy and paste relevant sections from manuscript text

내 답변

6b) Any changes to trial outcomes after the trial commenced, with reasons

Does your paper address CONSORT subitem 6b? *

Copy and paste relevant sections from the manuscript (include quotes in quotation marks "like this" to indicate direct quotes from your manuscript), or elaborate on this item by providing additional information not in the ms, or brie y explain why the item is not applicable/relevant for your study

No changes after start of trial.

7a) How sample size was determined

NPT: When applicable, details of whether and how the clustering by care provides or centers was addressed

7a-i) Describe whether and how expected attrition was taken into account when calculating the sample size

Describe whether and how expected attrition was taken into account when calculating the sample size.

	1	2	3	4	5	
subitem not at all important	○	○	○	○	○	essential

Does your paper address subitem 7a-i?

Copy and paste relevant sections from manuscript title (include quotes in quotation marks "like this" to indicate direct quotes from your manuscript), or elaborate on this item by providing additional information not in the ms, or brie y explain why the item is not applicable/relevant for your study

내 답변

7b) When applicable, explanation of any interim analyses and stopping guidelines

Does your paper address CONSORT subitem 7b? *

Copy and paste relevant sections from the manuscript (include quotes in quotation marks "like this" to indicate direct quotes from your manuscript), or elaborate on this item by providing additional information not in the ms, or brie y explain why the item is not applicable/relevant for your study

There were no interim analyses,as the study was conducted in its entirety without predefined stopping points or interim evaluations.

8a) Method used to generate the random allocation sequence

NPT: When applicable, how care providers were allocated to each trial group

Does your paper address CONSORT subitem 8a? *

Copy and paste relevant sections from the manuscript (include quotes in quotation marks "like this" to indicate direct quotes from your manuscript), or elaborate on this item by providing additional information not in the ms, or brie y explain why the item is not applicable/relevant for your study

"The random allocation sequence was generated using the clinical trial randomization tool (https://ctrandomization.cancer.gov/tool/) by an independent third party unrelated to the study, ensuring reproducibility. The randomization information was managed by an independent third party."

8b) Type of randomisation; details of any restriction (such as blocking and block size)

Does your paper address CONSORT subitem 8b? *

Copy and paste relevant sections from the manuscript (include quotes in quotation marks "like this" to indicate direct quotes from your manuscript), or elaborate on this item by providing additional information not in the ms, or brie y explain why the item is not applicable/relevant for your study

"After obtaining informed consent, the participants were assigned identification numbers and randomly allocated to either the VR or HFS training group using simple randomization."

9) Mechanism used to implement the random allocation sequence (such as sequentially numbered containers), describing any steps taken to conceal the sequence until interventions were assigned

Does your paper address CONSORT subitem 9? *

Copy and paste relevant sections from the manuscript (include quotes in quotation marks "like this" to indicate direct quotes from your manuscript), or elaborate on this item by providing additional information not in the ms, or brie y explain why the item is not applicable/relevant for your study

"After obtaining informed consent, the participants were assigned identification numbers and randomly allocated to either the VR or HFS training group using simple randomization. The random allocation sequence was generated using the clinical trial randomization tool (https://ctrandomization.cancer.gov/tool/) by an independent third party unrelated to the study, ensuring reproducibility. The randomization information was managed by an independent third party. All participant data were anonymized, and the trial administrators and personnel analyzing the data were blinded to the participants' identities and group assignments."

10) Who generated the random allocation sequence, who enrolled participants, and who assigned participants to interventions

Does your paper address CONSORT subitem 10? *

Copy and paste relevant sections from the manuscript (include quotes in quotation marks "like this" to indicate direct quotes from your manuscript), or elaborate on this item by providing additional information not in the ms, or brie y explain why the item is not applicable/relevant for your study

"In February 2024, 168 prospective interns who had recently graduated from medical school and were about to begin their internships were randomly divided into two groups for the RCT (Figure 1). After obtaining informed consent, the participants were assigned identification numbers and randomly allocated to either the VR or HFS training group using simple randomization. The random allocation sequence was generated using the clinical trial randomization tool (https://ctrandomization.cancer.gov/tool/) by an independent third party unrelated to the study, ensuring reproducibility. The randomization information was managed by an independent third party. All participant data were anonymized, and the trial administrators and personnel analyzing the data were blinded to the participants' identities and group assignments."

11a) If done, who was blinded after assignment to interventions (for example, participants, care providers, those assessing outcomes) and how

NPT: Whether or not administering co-interventions were blinded to group assignment

11a-i) Specify who was blinded, and who wasn't

Specify who was blinded, and who wasn't. Usually, in web-based trials it is not possible to blind the participants [1, 3] (this should be clearly acknowledged), but it may be possible to blind outcome assessors, those doing data analysis or those administering co-interventions (if any).

	1	2	3	4	5	
subitem not at all important	○	○	○	○	○	essential

Does your paper address subitem 11a-i? *

Copy and paste relevant sections from the manuscript (include quotes in quotation marks "like this" to indicate direct quotes from your manuscript), or elaborate on this item by providing additional information not in the ms, or brie y explain why the item is not applicable/relevant for your study

"After obtaining informed consent, the participants were assigned identification numbers and randomly allocated to either the VR or HFS training group using simple randomization. The random allocation sequence was generated using the clinical trial randomization tool (https://ctrandomization.cancer.gov/tool/) by an independent third party unrelated to the study, ensuring reproducibility. The randomization information was managed by an independent third party. All participant data were anonymized, and the trial administrators and personnel analyzing the data were blinded to the participants' identities and group assignments."

11a-ii) Discuss e.g., whether participants knew which intervention was the "intervention of interest" and which one was the "comparator"

Informed consent procedures (4a-ii) can create biases and certain expectations - discuss e.g., whether participants knew which intervention was the "intervention of interest" and which one was the "comparator".

	1	2	3	4	5	
subitem not at all important	○	○	○	○	○	essential

Does your paper address subitem 11a-ii?

Copy and paste relevant sections from the manuscript (include quotes in quotation marks "like this" to indicate direct quotes from your manuscript), or elaborate on this item by providing additional information not in the ms, or brie y explain why the item is not applicable/relevant for your study

내 답변

11b) If relevant, description of the similarity of interventions

(this item is usually not relevant for ehealth trials as it refers to similarity of a placebo or sham intervention to a active medication/intervention)

Does your paper address CONSORT subitem 11b? *

Copy and paste relevant sections from the manuscript (include quotes in quotation marks "like this" to indicate direct quotes from your manuscript), or elaborate on this item by providing additional information not in the ms, or brie y explain why the item is not applicable/relevant for your study

As this study involved comparing two distinct interventions (VR and HFS), there was no attempt to create similarity between the interventions.

12a) Statistical methods used to compare groups for primary and secondary outcomes

NPT: When applicable, details of whether and how the clustering by care providers or centers was addressed

Does your paper address CONSORT subitem 12a? *

Copy and paste relevant sections from the manuscript (include quotes in quotation marks "like this" to indicate direct quotes from your manuscript), or elaborate on this item by providing additional information not in the ms, or brie y explain why the item is not applicable/relevant for your study

"The answers from all questionnaires were compared between the VR and HFS groups. Statistical analysis was performed using the SPSS (version 25) statistical package (IBM SPSS Corp., Armonk, NY, USA). Continuous variables were analyzed with the t-test, and proportions were compared with the chi-square test. Changes in confidence before and after training, as well as in confidence according to the training method, were analyzed using a paired t-test, repeated-measures analysis of variance, and generalized estimating equations (GEEs). Effect sizes were calculated using the GEE and Cohen's d values. A p-value < 0.05 was considered significant."

12a-i) Imputation techniques to deal with attrition / missing values

Imputation techniques to deal with attrition / missing values: Not all participants will use the intervention/comparator as intended and attrition is typically high in ehealth trials. Specify how participants who did not use the application or dropped out from the trial were treated in the statistical analysis (a complete case analysis is strongly discouraged, and simple imputation techniques such as LOCF may also be problematic [4]).

	1	2	3	4	5	
subitem not at all important	○	○	○	○	○	essential

Does your paper address subitem 12a-i? *

Copy and paste relevant sections from the manuscript (include quotes in quotation marks "like this" to indicate direct quotes from your manuscript), or elaborate on this item by providing additional information not in the ms, or brie y explain why the item is not applicable/relevant for your study

"This study included 168 prospective interns who were randomly assigned to either the VR group (n = 81) or the HFS group (n = 87). No dropouts were reported before randomization. Data from four participants who did not complete the pre-survey were excluded from the final analysis, resulting in 79 participants in the VR group and 85 in the HFS group."

12b) Methods for additional analyses, such as subgroup analyses and adjusted analyses

Does your paper address CONSORT subitem 12b? *

Copy and paste relevant sections from the manuscript (include quotes in quotation marks "like this" to indicate direct quotes from your manuscript), or elaborate on this item by providing additional information not in the ms, or brie y explain why the item is not applicable/relevant for your study

"As a training method, VR simulation was preferred (63/139, 45.3%) over HFS simulation (54/139, 38.8%) and lectures (22/139, 15.8%). A higher proportion of participants in the VR group preferred VR, while a higher proportion in the HFS group preferred HFS. Despite not assigning participants based on their training method preferences, the observed difference in preference between the groups was likely due to the influence of the group they were assigned to, as the survey was conducted after the random assignment. The alignment between the participants' preferred training method and the method they followed did not significantly affect satisfaction ($t(137) = 0.21$, $P = .13$) or the increase in confidence following the training ($F (1, 137) = 0.08$, $P = .45$) .... The average (SD) time spent on the simple case was 151.05 (60.94) seconds, whereas 132.95 (67.58) seconds was spent on the complex case, with no significant difference between the two cases ($t(69) = 1.17$, $P = .11$). The average number of tasks completed out of the six subtasks was 3.19 in the simple case and 4.33 in the complex case ($t(69) = −3.02$, $P = .002$). Correlation analysis revealed no significant relationships between the time spent on each scenario, the number of tasks completed, participant satisfaction with the training, and their confidence to handle the situation (Multimedia appendix 3). Multiple regression analysis was conducted to examine whether factors such as the time taken by each intern to solve problems and the number of tasks cleared, derived from log data during the VR training sessions, influenced the improvement in confidence levels after the class. However, none of the factors had a significant impact on post-training confidence levels, except pre-training confidence."

X26) REB/IRB Approval and Ethical Considerations [recommended as subheading under "Methods"] (not a CONSORT item)

X26-i) Comment on ethics committee approval

	1	2	3	4	5	
subitem not at all important	○	○	○	○	○	essential

Does your paper address subitem X26-i?

Copy and paste relevant sections from the manuscript (include quotes in quotation marks "like this" to indicate direct quotes from your manuscript), or elaborate on this item by providing additional information not in the ms, or brie y explain why the item is not applicable/relevant for your study

내 답변

x26-ii) Outline informed consent procedures

Outline informed consent procedures e.g., if consent was obtained o ine or online (how? Checkbox, etc.?), and what information was provided (see 4a-ii). See [6] for some items to be included in informed consent documents.

	1	2	3	4	5	
subitem not at all important	○	○	○	○	○	essential

Does your paper address subitem X26-ii?

Copy and paste relevant sections from the manuscript (include quotes in quotation marks "like this" to indicate direct quotes from your manuscript), or elaborate on this item by providing additional information not in the ms, or brie y explain why the item is not applicable/relevant for your study

내 답변

X26-iii) Safety and security procedures

Safety and security procedures, incl. privacy considerations, and any steps taken to reduce the likelihood or detection of harm (e.g., education and training, availability of a hotline)

	1	2	3	4	5	
subitem not at all important	○	○	○	○	○	essential

Does your paper address subitem X26-iii?

Copy and paste relevant sections from the manuscript (include quotes in quotation marks "like this" to indicate direct quotes from your manuscript), or elaborate on this item by providing additional information not in the ms, or brie y explain why the item is not applicable/relevant for your study

내 답변

RESULTS

13a) For each group, the numbers of participants who were randomly assigned, received intended treatment, and were analysed for the primary outcome

NPT: The number of care providers or centers performing the intervention in each group and the number of patients treated by each care provider in each center

Does your paper address CONSORT subitem 13a? *

Copy and paste relevant sections from the manuscript (include quotes in quotation marks "like this" to indicate direct quotes from your manuscript), or elaborate on this item by providing additional information not in the ms, or brie y explain why the item is not applicable/relevant for your study

"This study included 168 prospective interns who were randomly assigned to either the VR group (n = 81) or the HFS group (n = 87). No dropouts were reported before randomization."

13b) For each group, losses and exclusions after randomisation, together with reasons

Does your paper address CONSORT subitem 13b? (NOTE: Preferably, this is shown in a CONSORT flow diagram) *

Copy and paste relevant sections from the manuscript (include quotes in quotation marks "like this" to indicate direct quotes from your manuscript), or elaborate on this item by providing additional information not in the ms, or brie y explain why the item is not applicable/relevant for your study

"Data from four participants who did not complete the pre-survey were excluded from the final analysis, resulting in 79 participants in the VR group and 85 in the HFS group."

13b-i) Attrition diagram

Strongly recommended: An attrition diagram (e.g., proportion of participants still logging in or using the intervention/comparator in each group plotted over time, similar to a survival curve) or other gures or tables demonstrating usage/dose/engagement.

	1	2	3	4	5	
subitem not at all important	○	○	○	○	○	essential

Does your paper address subitem 13b-i?

Copy and paste relevant sections from the manuscript or cite the gure number if applicable (include quotes in quotation marks "like this" to indicate direct quotes from your manuscript), or elaborate on this item by providing additional information not in the ms, or brie y explain why the item is not applicable/relevant for your study

내 답변

14a) Dates defining the periods of recruitment and follow-up

Does your paper address CONSORT subitem 14a? *

Copy and paste relevant sections from the manuscript (include quotes in quotation marks "like this" to indicate direct quotes from your manuscript), or elaborate on this item by providing additional information not in the ms, or brie y explain why the item is not applicable/relevant for your study

"In February 2024, 168 prospective interns who had recently graduated from medical school and were about to begin their internships were randomly divided into two groups for the RCT (Figure 1) To further evaluate the effect of the program, FGIs were conducted with the interns and tutors 1 and 5 months post-training."

14a-i) Indicate if critical "secular events" fell into the study period

Indicate if critical "secular events" fell into the study period, e.g., signi cant changes in Internet resources available or "changes in computer hardware or Internet delivery resources"

	1	2	3	4	5	
subitem not at all important	○	○	○	○	○	essential

Does your paper address subitem 14a-i?

Copy and paste relevant sections from the manuscript (include quotes in quotation marks "like this" to indicate direct quotes from your manuscript), or elaborate on this item by providing additional information not in the ms, or brie y explain why the item is not applicable/relevant for your study

내 답변

14b) Why the trial ended or was stopped (early)

Does your paper address CONSORT subitem 14b? *

Copy and paste relevant sections from the manuscript (include quotes in quotation marks "like this" to indicate direct quotes from your manuscript), or elaborate on this item by providing additional information not in the ms, or brie y explain why the item is not applicable/relevant for your study

There was no early trial end.

15) A table showing baseline demographic and clinical characteristics for each group

NPT: When applicable, a description of care providers (case volume, quali cation, expertise, etc.) and centers (volume) in each group

Does your paper address CONSORT subitem 15? *

Copy and paste relevant sections from the manuscript (include quotes in quotation marks "like this" to indicate direct quotes from your manuscript), or elaborate on this item by providing additional information not in the ms, or brie y explain why the item is not applicable/relevant for your study

"Data from four participants who did not complete the pre-survey were excluded from the final analysis, resulting in 79 participants in the VR group and 85 in the HFS group. The sex ratio (male: female) was 1.9:1 in the HFS group and 1.4:1 in the VR group, with no significant difference between the groups (P = .34). The mean age did not differ between the groups (P = .29) (Multimedia appendix 2)."

15-i) Report demographics associated with digital divide issues

In ehealth trials it is particularly important to report demographics associated with digital divide issues, such as age, education, gender, social-economic status, computer/Internet/ehealth literacy of the participants, if known.

	1	2	3	4	5	
subitem not at all important	○	○	○	○	○	essential

Does your paper address subitem 15-i? *

Copy and paste relevant sections from the manuscript (include quotes in quotation marks "like this" to indicate direct quotes from your manuscript), or elaborate on this item by providing additional information not in the ms, or brie y explain why the item is not applicable/relevant for your study

"The sex ratio (male: female) was 1.9:1 in the HFS group and 1.4:1 in the VR group, with no significant difference between the groups (P = .34). The mean age did not differ between the groups (P = .29) (Multimedia appendix 2)."

16) For each group, number of participants (denominator) included in each analysis and whether the analysis was by original assigned groups

16-i) Report multiple “denominators” and provide definitions

Report multiple “denominators” and provide de nitions: Report N’s (and effect sizes) “across a range of study participation [and use] thresholds” [1], e.g., N exposed, N consented, N used more than x times, N used more than y weeks, N participants “used” the intervention/comparator at speci c pre-de ned time points of interest (in absolute and relative numbers per group). Always clearly de ne “use” of the intervention.

	1	2	3	4	5	
subitem not at all important	○	○	○	○	○	essential

Does your paper address subitem 16-i? *

Copy and paste relevant sections from the manuscript (include quotes in quotation marks "like this" to indicate direct quotes from your manuscript), or elaborate on this item by providing additional information not in the ms, or brie y explain why the item is not applicable/relevant for your study

"This study included 168 prospective interns who were randomly assigned to either the VR group (n = 81) or the HFS group (n = 87). No dropouts were reported before randomization. Data from four participants who did not complete the pre-survey were excluded from the final analysis, resulting in 79 participants in the VR group and 85 in the HFS group."

16-ii) Primary analysis should be intent-to-treat

Primary analysis should be intent-to-treat, secondary analyses could include comparing only “users”, with the appropriate caveats that this is no longer a randomized sample (see 18-i).

	1	2	3	4	5	
subitem not at all important	○	○	○	○	○	essential

Does your paper address subitem 16-ii?

Copy and paste relevant sections from the manuscript (include quotes in quotation marks "like this" to indicate direct quotes from your manuscript), or elaborate on this item by providing additional information not in the ms, or brie y explain why the item is not applicable/relevant for your study

내 답변

17a) For each primary and secondary outcome, results for each group, and the estimated effect size and its precision (such as 95% confidence interval)

Does your paper address CONSORT subitem 17a? *

Copy and paste relevant sections from the manuscript (include quotes in quotation marks "like this" to indicate direct quotes from your manuscript), or elaborate on this item by providing additional information not in the ms, or brie y explain why the item is not applicable/relevant for your study

"The mean (SD) confidence score for managing a patient with low oxygen saturation before training, on a 10-point scale, was 3.77 (2.12) but increased significantly to 6.20 (2.02) after training ($t(163) = -14.04$, $P < .001$). Notably, confidence in handling unrelated scenarios, such as seizures, anaphylaxis, general care, and basic skills, which were not directly covered during the training, also increased significantly after the training, indicating a broader impact of the "desaturation management" training. However, the increase in confidence for managing oxygen desaturation was significantly higher compared to that for the other clinical situations ($t(163) = -13.87$, $P < .001$, Cohen's $d = -1.08$).
Table 1 presents an analysis of confidence in the HFS and VR groups when managing changes in a desaturated patient, before and after training. The mean (SD) confidence scores increased significantly from 3.82 (2.31) to 6.54 (1.91) in the HFS group ($t(84) = -10.79$, $P < .001$) and from 3.72 (1.91) to 5.82 (2.08) in the VR group ($t(78) = -9.15$, $P < .001$), but the type of training method did not significantly affect confidence ($F (1, 162) = 3.28$, $P = .07$), implying that VR is not inferior to HFS in this context. Similarly, confidence in managing other clinical situations not directly covered in the training also improved after the program. However, no significant difference was observed between the training methods."

17a-i) Presentation of process outcomes such as metrics of use and intensity of use

In addition to primary/secondary (clinical) outcomes, the presentation of process outcomes such as metrics of use and intensity of use (dose, exposure) and their operational de nitions is critical. This does not only refer to metrics of attrition (13-b) (often a binary variable), but also to more continuous exposure metrics such as "average session length". These must be accompanied by a technical description how a metric like a "session" is de ned (e.g., timeout after idle time) [1] (report under item 6a).

	1	2	3	4	5	
subitem not at all important	○	○	○	○	○	essential

Does your paper address subitem 17a-i?

Copy and paste relevant sections from the manuscript (include quotes in quotation marks "like this" to indicate direct quotes from your manuscript), or elaborate on this item by providing additional information not in the ms, or brie y explain why the item is not applicable/relevant for your study

내 답변

17b) For binary outcomes, presentation of both absolute and relative effect sizes is recommended

Does your paper address CONSORT subitem 17b? *

Copy and paste relevant sections from the manuscript (include quotes in quotation marks "like this" to indicate direct quotes from your manuscript), or elaborate on this item by providing additional information not in the ms, or brie y explain why the item is not applicable/relevant for your study

There were no binary outcomes.

18) Results of any other analyses performed, including subgroup analyses and adjusted analyses, distinguishing pre-specified from exploratory

Does your paper address CONSORT subitem 18? *

Copy and paste relevant sections from the manuscript (include quotes in quotation marks "like this" to indicate direct quotes from your manuscript), or elaborate on this item by providing additional information not in the ms, or brie y explain why the item is not applicable/relevant for your study

"As a training method, VR simulation was preferred (63/139, 45.3%) over HFS simulation (54/139, 38.8%) and lectures (22/139, 15.8%). A higher proportion of participants in the VR group preferred VR, while a higher proportion in the HFS group preferred HFS. Despite not assigning participants based on their training method preferences, the observed difference in preference between the groups was likely due to the influence of the group they were assigned to, as the survey was conducted after the random assignment. The alignment between the participants' preferred training method and the method they followed did not significantly affect satisfaction (t(137) = 0.21, P = .13) or the increase in confidence following the training (F (1, 137) = 0.08, P = .45) .... The average (SD) time spent on the simple case was 151.05 (60.94) seconds, whereas 132.95 (67.58) seconds was spent on the complex case, with no significant difference between the two cases (t(69) = 1.17, P = .11). The average number of tasks completed out of the six subtasks was 3.19 in the simple case and 4.33 in the complex case (t(69) = −3.02, P = .002). Correlation analysis revealed no significant relationships between the time spent on each scenario, the number of tasks completed, participant satisfaction with the training, and their confidence to handle the situation (Multimedia appendix 3). Multiple regression analysis was conducted to examine whether factors such as the time taken by each intern to solve problems and the number of tasks cleared, derived from log data during the VR training sessions, influenced the improvement in confidence levels after the class. However, none of the factors had a significant impact on post-training confidence levels, except pre-training confidence."

18-i) Subgroup analysis of comparing only users

A subgroup analysis of comparing only users is not uncommon in ehealth trials, but if done, it must be stressed that this is a self-selected sample and no longer an unbiased sample from a randomized trial (see 16-iii).

	1	2	3	4	5	
subitem not at all important	○	○	○	○	○	essential

Does your paper address subitem 18-i?

Copy and paste relevant sections from the manuscript (include quotes in quotation marks "like this" to indicate direct quotes from your manuscript), or elaborate on this item by providing additional information not in the ms, or brie y explain why the item is not applicable/relevant for your study

내 답변

19) All important harms or unintended effects in each group

(for speci c guidance see CONSORT for harms)

Does your paper address CONSORT subitem 19? *

Copy and paste relevant sections from the manuscript (include quotes in quotation marks "like this" to indicate direct quotes from your manuscript), or elaborate on this item by providing additional information not in the ms, or brie y explain why the item is not applicable/relevant for your study

There were no harms or unintended effects.

19-i) Include privacy breaches, technical problems

Include privacy breaches, technical problems. This does not only include physical “harm” to participants, but also incidents such as perceived or real privacy breaches [1], technical problems, and other unexpected/unintended incidents. “Unintended effects” also includes unintended positive effects [2].

	1	2	3	4	5	
subitem not at all important	○	○	○	○	○	essential

Does your paper address subitem 19-i?

Copy and paste relevant sections from the manuscript (include quotes in quotation marks "like this" to indicate direct quotes from your manuscript), or elaborate on this item by providing additional information not in the ms, or brie y explain why the item is not applicable/relevant for your study

내 답변

19-ii) Include qualitative feedback from participants or observations from staff/researchers

Include qualitative feedback from participants or observations from staff/researchers, if available, on strengths and shortcomings of the application, especially if they point to unintended/unexpected effects or uses. This includes (if available) reasons for why people did or did not use the application as intended by the developers.

	1	2	3	4	5	
subitem not at all important	○	○	○	○	○	essential

Does your paper address subitem 19-ii?

Copy and paste relevant sections from the manuscript (include quotes in quotation marks "like this" to indicate direct quotes from your manuscript), or elaborate on this item by providing additional information not in the ms, or brie y explain why the item is not applicable/relevant for your study

내 답변

DISCUSSION

22) Interpretation consistent with results, balancing benefits and harms, and considering other relevant evidence

NPT: In addition, take into account the choice of the comparator, lack of or partial blinding, and unequal expertise of care providers or centers in each group

22-i) Restate study questions and summarize the answers suggested by the data, starting with primary outcomes and process outcomes (use)

Restate study questions and summarize the answers suggested by the data, starting with primary outcomes and process outcomes (use).

	1	2	3	4	5	
subitem not at all important	○	○	○	○	○	essential

Does your paper address subitem 22-i? *

Copy and paste relevant sections from the manuscript (include quotes in quotation marks "like this" to indicate direct quotes from your manuscript), or elaborate on this item by providing additional information not in the ms, or brie y explain why the item is not applicable/relevant for your study

"This study investigated the effectiveness of VR simulation training compared to HFS training in preparing medical interns for emergencies, specifically focusing on scenarios involving managing patients with decreasing oxygen saturation. Our findings indicate that VR and HFS significantly improved the confidence and preparedness of the interns, with no substantial difference in the overall confidence level between the groups. Pre-training preference favored VR education, although post-training satisfaction was significantly higher in the simulator education group.
In this study, the significant increase in self-confidence when managing emergency scenarios in the VR and HFS training groups underscored the value of simulation-based education. VR training demonstrated educational effectiveness similar to that of HFS, which was previously established [18-20]. Immersive VR simulation education reproduces challenging clinical scenarios more easily than does high-fidelity simulators [21-23]. However, participants perceived that the actual medical equipment used in simulator education, such as transfer monitors, oxygen tanks, and transfer carts, provided a greater sense of realism than interactions in VR.
Although overall satisfaction and the sense of experiencing actual clinical situations were greater with simulator education in this study, no difference in confidence was observed based on the educational method. Qualitative feedback from the FGIs provided further insights into the strengths and weaknesses of VR training. The interns reported that the VR training was highly effective in helping them to stay composed and to manage similar situations in real clinical settings. They found VR particularly useful in scenarios in which they needed to respond independently, as the realistic implementation of warning sounds from various monitors and the requirement to handle situations alone, as outlined in the scenarios, closely mirrored real-life experiences. However, the participants reported that it would be even more beneficial to have the opportunity to physically see and interact with the actual equipment, such as oxygen flowmeters, oxygen tanks, and monitors. These aspects could potentially be improved by implementing VR with greater realism or integrating mixed reality with augmented reality features, as well as utilizing haptic devices.
VR education offers the distinct advantage of allowing each participant to take on an active, central role in a simulation. However, it also presents challenges, particularly in the areas of debriefing and feedback, which are crucial components of simulation-based education. Unlike more established methods, the approaches for debriefing in VR settings are still evolving. As VR simulation becomes increasingly prevalent, educators are exploring and experimenting with various alternatives to traditional debriefing methods. These include in-person debriefing, virtual debriefing, and self-debriefing. In this study, debriefing was conducted in person, with careful consideration given to ensure consistent group sizes and equitable time allocation. However, a unique challenge arose with the VR simulations, in which participants were unable to mirror the simulations simultaneously when using HMDs. In post-education FGIs with the educators, HFS facilitated easier debriefing and feedback compared to the VR simulations, which face limitations. Interestingly, the learners did not express any negative opinions about the VR debriefing process in their FGIs. This discrepancy could be due to educators having experience in both types of education, suggesting a need for further research, particularly as existing studies offer mixed results regarding debriefing outcomes in VR education [24]."

22-ii) Highlight unanswered new questions, suggest future research

Highlight unanswered new questions, suggest future research.

	1	2	3	4	5	
subitem not at all important	○	○	○	○	○	essential

Does your paper address subitem 22-ii?

Copy and paste relevant sections from the manuscript (include quotes in quotation marks "like this" to indicate direct quotes from your manuscript), or elaborate on this item by providing additional information not in the ms, or brie y explain why the item is not applicable/relevant for your study

내 답변

20) Trial limitations, addressing sources of potential bias, imprecision, and, if relevant, multiplicity of analyses

20-i) Typical limitations in ehealth trials

Typical limitations in ehealth trials: Participants in ehealth trials are rarely blinded. Ehealth trials often look at a multiplicity of outcomes, increasing risk for a Type I error. Discuss biases due to non-use of the intervention/usability issues, biases through informed consent procedures, unexpected events.

	1	2	3	4	5	
subitem not at all important	○	○	○	○	○	essential

Does your paper address subitem 20-i? *

Copy and paste relevant sections from the manuscript (include quotes in quotation marks "like this" to indicate direct quotes from your manuscript), or elaborate on this item by providing additional information not in the ms, or brie y explain why the item is not applicable/relevant for your study

"This study had several limitations. First, in comparing the effectiveness of the different educational methods, this study assessed self-efficacy rather than objectively measuring performance differences between the groups. Given that the emergency scenarios addressed in this study were relatively straightforward, we considered evaluating the interns' responses in simulated situations, such as an objective structured clinical exam. However, the primary goal of this training was to prepare the interns to respond effectively when encountering similar situations during their internship. Therefore, we measured changes in self-efficacy during their internship. While this approach provided valuable insight beyond merely assessing satisfaction levels, future research should examine differences in actual performance in short- and long-term studies. Second, there were inconsistencies in the level of feedback provided during the debriefing stage. Direct feedback on actual performance was not given in the VR group, which could be addressed in future studies by incorporating techniques such as mirroring to ensure more immediate and targeted feedback. Third, this study did not account for participants' previous experience with VR. Although most students likely had some familiarity with VR, collecting more precise data would enhance the accuracy of the findings."

21) Generalisability (external validity, applicability) of the trial findings

NPT: External validity of the trial ndings according to the intervention, comparators, patients, and care providers or centers involved in the trial

21-i) Generalizability to other populations

Generalizability to other populations: In particular, discuss generalizability to a general Internet population, outside of a RCT setting, and general patient population, including applicability of the study results for other organizations

	1	2	3	4	5	
subitem not at all important	○	○	○	○	○	essential

Does your paper address subitem 21-i?

Copy and paste relevant sections from the manuscript (include quotes in quotation marks "like this" to indicate direct quotes from your manuscript), or elaborate on this item by providing additional information not in the ms, or brie y explain why the item is not applicable/relevant for your study

내 답변

21-ii) Discuss if there were elements in the RCT that would be different in a routine application setting

Discuss if there were elements in the RCT that would be different in a routine application setting (e.g., prompts/reminders, more human involvement, training sessions or other co-interventions) and what impact the omission of these elements could have on use, adoption, or outcomes if the intervention is applied outside of a RCT setting.

	1	2	3	4	5	
subitem not at all important	○	○	○	○	○	essential

Does your paper address subitem 21-ii?

Copy and paste relevant sections from the manuscript (include quotes in quotation marks "like this" to indicate direct quotes from your manuscript), or elaborate on this item by providing additional information not in the ms, or brie y explain why the item is not applicable/relevant for your study

내 답변

OTHER INFORMATION

23) Registration number and name of trial registry

Does your paper address CONSORT subitem 23? *

Copy and paste relevant sections from the manuscript (include quotes in quotation marks "like this" to indicate direct quotes from your manuscript), or elaborate on this item by providing additional information not in the ms, or brie y explain why the item is not applicable/relevant for your study

Trial registration: ClinicalTrials.gov NCT06295887

24) Where the full trial protocol can be accessed, if available

Does your paper address CONSORT subitem 24? *

Cite a Multimedia Appendix, other reference, or copy and paste relevant sections from the manuscript (include quotes in quotation marks "like this" to indicate direct quotes from your manuscript), or elaborate on this item by providing additional information not in the ms, or brie y explain why the item is not applicable/relevant for your study

"The CONSORT Checklist and the full trial protocol can be accessed in Multimedia appendix 1."

25) Sources of funding and other support (such as supply of drugs), role of funders

Does your paper address CONSORT subitem 25? *

Copy and paste relevant sections from the manuscript (include quotes in quotation marks "like this" to indicate direct quotes from your manuscript), or elaborate on this item by providing additional information not in the ms, or brie y explain why the item is not applicable/relevant for your study

There were no funders for this study.

X27) Conflicts of Interest (not a CONSORT item)

X27-i) State the relation of the study team towards the system being evaluated

In addition to the usual declaration of interests (nancial or otherwise), also state the relation of the study team towards the system being evaluated, i.e., state if the authors/evaluators are distinct from or identical with the developers/sponsors of the intervention.

	1	2	3	4	5	
subitem not at all important	○	○	○	○	○	essential

Does your paper address subitem X27-i?

Copy and paste relevant sections from the manuscript (include quotes in quotation marks "like this" to indicate direct quotes from your manuscript), or elaborate on this item by providing additional information not in the ms, or brie y explain why the item is not applicable/relevant for your study

내 답변

About the CONSORT EHEALTH checklist

As a result of using this checklist, did you make changes in your manuscript? *

- ○ yes, major changes
- ◉ yes, minor changes
- ○ no

What were the most important changes you made as a result of using this checklist?

내 답변

How much time did you spend on going through the checklist INCLUDING making changes in your manuscript *

Appoximately 7 to 8 hours.

As a result of using this checklist, do you think your manuscript has improved? *

- ( ) yes
- (•) no
- ( ) 기타:

Would you like to become involved in the CONSORT EHEALTH group?

This would involve for example becoming involved in participating in a workshop and writing an "Explanation and Elaboration" document

- ( ) yes
- ( ) no
- ( ) 기타:

Any other comments or questions on CONSORT EHEALTH

내 답변

STOP - Save this form as PDF before you click submit

To generate a record that you lled in this form, we recommend to generate a PDF of this page (on a Mac, simply select "print" and then select "print as PDF") before you submit it.

When you submit your (revised) paper to JMIR, please upload the PDF as supplementary le.

Don't worry if some text in the textboxes is cut off, as we still have the complete information in our database. Thank you!

Final step: Click submit !

Click submit so we have your answers in our database!

제출 양식 지우기

Google Forms를 통해 비밀번호를 제출하지 마세요.

이 콘텐츠는 Google이 만들거나 승인하지 않았습니다. - 서비스 약관 - 개인정보처리방침

Does this form look suspicious? 보고서

Google 설문지

<Clinical Trial Protocol>

Title of the Study:
Immersive Virtual Reality Training to Improve Novice Physicians' Emergency Response Skills: A Randomized Controlled Trial
Study Design:
This is a two-arm parallel randomized controlled trial (RCT) comparing the effectiveness of Virtual Reality (VR) and High-Fidelity Simulation (HFS) in improving emergency response skills among medical interns.
Primary Objectives:

- To evaluate the improvement in confidence for managing patients with low oxygen saturation after VR or HFS training.
- To compare the participant satisfaction with the VR and HFS training methods.

Secondary Objectives:

- To assess the usability of the VR and HFS systems.
- To explore real-world applicability and behavioral changes through focus group inte rviews (FGIs).

Study Population:
Medical interns enrolled in the internship program at Seoul National University Hospital.
Inclusion Criteria:

- Medical interns who recently graduated from medical school.
- Participants who provided written informed consent.

Exclusion Criteria:

- Interns who did not complete the pre-training survey.

Intervention:

- **VR Group**: Participants used a VR-based training program to simulate managing pat ients with low oxygen saturation.
- **HFS Group**: Participants used an HFS-based training program for the same scenario.

Outcome Measures:

- **Primary Outcomes**:
  - Confidence improvement (measured using a 10-point Likert scale).
  - Participant satisfaction (measured using a 7-point Likert scale).
- **Secondary Outcomes**:
  - Usability assessment using the User Experience Questionnaire Short (UEQ-S).
  - Behavioral changes assessed through FGIs conducted 1 and 5 months after tr aining.

Randomization:
Participants were randomly assigned to VR or HFS groups using a clinical trial randomization tool. An independent third party generated and managed the randomization sequence.
Blinding:
The researchers analyzing the data were blinded to the group assignments to ensure unbiased analysis.

Timeline:

- Recruitment and pre-training data collection: February 2024
- Training sessions: February 2024
- Post-training assessments: February 2024
- FGIs: March and July 2024
